# Perivascular Arrest of CD8^+^ T Cells Is a Signature of Experimental Cerebral Malaria

**DOI:** 10.1371/journal.ppat.1005210

**Published:** 2015-11-12

**Authors:** Tovah N. Shaw, Phillip J. Stewart-Hutchinson, Patrick Strangward, Durga B. Dandamudi, Jonathan A. Coles, Ana Villegas-Mendez, Julio Gallego-Delgado, Nico van Rooijen, Egor Zindy, Ana Rodriguez, James M. Brewer, Kevin N. Couper, Michael L. Dustin

**Affiliations:** 1 Faculty of Life Sciences, University of Manchester, Manchester, United Kingdom; 2 Molecular Pathogenesis Program, Helen L. and Martin S. Kimmel Center for Biology and Medicine, Skirball Institute of Biomolecular Medicine, New York, New York, United States of America; 3 Department of Pediatric Research, Washington University School of Medicine, St. Louis, Missouri, United States of America; 4 Immunology and Inflammation Program, Helen L. and Martin S. Kimmel Center for Biology and Medicine, Skirball Institute of Biomolecular Medicine, New York, New York, United States of America; 5 Centre for Immunobiology, Institute of Infection, Immunity and Inflammation, College of Medical, Veterinary and Life Sciences, University of Glasgow, Glasgow, United Kingdom; 6 Department of Microbiology, New York University School of Medicine, New York, New York, United States of America; 7 Department of Molecular Cell Biology, VU Medical Center, Amsterdam, The Netherlands; 8 The Wellcome Trust Centre for Cell-Matrix Research, Faculty of Life Sciences, University of Manchester, Manchester, United Kingdom; 9 The Kennedy Institute of Rheumatology, Nuffield Department of Orthopaedics and Musculoskeletal Sciences, The University of Oxford, Headington, United Kingdom; Queensland Institute of Medical Research, AUSTRALIA

## Abstract

There is significant evidence that brain-infiltrating CD8^+^ T cells play a central role in the development of experimental cerebral malaria (ECM) during *Plasmodium berghei* ANKA infection of C57BL/6 mice. However, the mechanisms through which they mediate their pathogenic activity during malaria infection remain poorly understood. Utilizing intravital two-photon microscopy combined with detailed *ex vivo* flow cytometric analysis, we show that brain-infiltrating T cells accumulate within the perivascular spaces of brains of mice infected with both ECM-inducing (*P*. *berghei* ANKA) and non-inducing (*P*. *berghei* NK65) infections. However, perivascular T cells displayed an arrested behavior specifically during *P*. *berghei* ANKA infection, despite the brain-accumulating CD8^+^ T cells exhibiting comparable activation phenotypes during both infections. We observed T cells forming long-term cognate interactions with CX_3_CR1-bearing antigen presenting cells within the brains during *P*. *berghei* ANKA infection, but abrogation of this interaction by targeted depletion of the APC cells failed to prevent ECM development. Pathogenic CD8^+^ T cells were found to colocalize with rare apoptotic cells expressing CD31, a marker of endothelial cells, within the brain during ECM. However, cellular apoptosis was a rare event and did not result in loss of cerebral vasculature or correspond with the extensive disruption to its integrity observed during ECM. In summary, our data show that the arrest of T cells in the perivascular compartments of the brain is a unique signature of ECM-inducing malaria infection and implies an important role for this event in the development of the ECM-syndrome.

## Author Summary

Cerebral malaria is the most severe complication of *Plasmodium falciparum* infection. Utilizing the murine experimental model of cerebral malaria (ECM), it has been found that CD8^+^ T cells are a key immune cell type responsible for development of cerebral pathology during malaria infection. To identify how CD8^+^ T cells cause cerebral pathology during malaria infection, in this study we have performed detailed *in vivo* analysis (two photon imaging) of CD8^+^ T cells within the brains of mice infected with strains of malaria parasites that cause or do not cause ECM. We found that CD8^+^ T cells appear to accumulate in similar numbers and in comparable locations within the brains of mice infected with parasites that do or do not cause ECM. Importantly, however, brain accumulating CD8^+^ T cells displayed significantly different movement characteristics during the different infections. CD8^+^ T cells interacted with myeloid cells within the brain during infection with parasites causing ECM, but this association was not required for development of cerebral complications. Furthermore, our results suggest that CD8^+^ T cells do not cause ECM through the widespread killing of brain microvessel cells. The results in this study significantly improve our understanding of the ways through which CD8^+^ T cells can mediate cerebral pathology during malaria infection.

## Introduction

Malaria remains a significant global health problem with 207 million cases, resulting in 584,000–1,238,000 deaths, annually [1, 2]. A high proportion of these deaths are due to cerebral malaria (CM), a neuropathology induced primarily by the species *Plasmodium falciparum* [2]. Current treatment of cerebral malaria is limited to parasiticidal chemotherapies, typically administered late in the course of infection. These traditional and narrowly targeted interventions are ineffective in many cases, and the mortality rate of CM, even after treatment, remains at 10–20% [3–5]. A greater understanding of the parasitological and immunological events leading to the development of CM would aid the development of improved therapeutic options to treat the condition.

Infection of susceptible strains of mice with *Plasmodium berghei* ANKA (*Pb* ANKA) results in the development of a serious neurological syndrome, termed experimental cerebral malaria (ECM), which recapitulates many of the clinical and pathological features of CM [6–10]. Susceptible mice typically develop neurological signs of disease including ataxia, convulsions, paralysis and coma between 6 and 8 days post infection [7, 11]. Histologically visible hemorrhages, widespread disruption of the vascular integrity and accumulation of leukocyte subsets are observed within the brain concomitant with the onset of signs of disease, [12–14]. The reason why *Pb* ANKA causes ECM while other strains of *P*. *berghei*, such as *P*. *berghei* NK65, do not is an area of active investigation. However, the differing virulence of *P*. *berghei* parasites does not appear to be due to extensive genetic polymorphisms between strains [15, 16].

Multiple cell types, including monocytes, macrophages, NK cells and CD8^+^ T cells accumulate within the brain at the onset of ECM [17–20]. However, to date, only CD8^+^ T cells have been identified as playing an unequivocal role in the development of cerebral pathology; protection from ECM is afforded by their depletion as late as one day prior to the development of neurological signs [10, 12, 19, 21]. The pathogenic parasite-specific CD8^+^ T cells are primed in the spleen by CD8α^+^ dendritic cells (DCs) [22] before migrating to the brain through homing dependent upon IFNγ-stimulated CXCL10 production in the CNS [23]. Monocytes play a role in recruitment of the pathogenic CD8^+^ T cells to the brain during ECM; however, the relative importance of this event in development of cerebral pathology remains undefined [19]. It has previously been shown that parasite-specific CD8^+^ T cells mediate ECM development through perforin- and granzyme B-dependent mechanisms [11, 24, 25], yet where the CD8^+^ T cells localize within the brain to cause ECM has remained unclear.

Parasite-specific CD8^+^ T cells appear to require *in situ* antigen-dependent stimulation within the brain to program their pathogenic activity necessary for ECM development [11]. To date, however, the identity of the putative antigen cross-presenting cells that interact with pathogenic CD8^+^ T cells during ECM is unknown. Recently, it has been shown that parasite specific CD8^+^ T cells can specifically interact with antigen cross-presenting microvessel cells obtained from mice experiencing ECM [26], but the relevance of this interaction for development of ECM *in vivo* is undefined.

In other models of neuroinflammatory diseases, such as experimental autoimmune encephalomyelitis (EAE), it has been demonstrated that professional antigen presenting cells (APCs) within the subarachnoid (SA) and perivascular spaces of the central nervous system (CNS) present antigen to T cells, instructing their pathogenic function [27–31]. Whether interaction of CD8^+^ T cells with brain-resident or infiltrating APC types is a canonical event in ECM development is largely unexplored and may represent a hitherto unexplored mechanism in the development of ECM.

In this study, we have attempted to reveal, *in vivo*, the mechanisms through which brain-infiltrating CD8^+^ T cells cause ECM. Using transcranial intravital two-photon microscopy, we report that T cells are recruited to, and accumulate perivascularly within, the SA and perivascular spaces of mice infected with both ECM-inducing and non-ECM-inducing *Plasmodium berghei* strains. However, a high proportion of perivascular T cells exhibited arrested behavior, consistent with immunological synapse formation [32–34], in the meninges specifically during ECM-inducing malaria infection. These arrested perivascular T cells formed cognate interactions with cells expressing CX_3_CR1, comprising inflammatory monocytes, macrophages and dendritic cells, but this event was redundant for ECM development. Pathogenic CD8^+^ T cells co-localized with apoptotic CD31^+^ cells in brains of mice with ECM, but apoptosis was a rare event in relation to the extensive vascular leakage observed during ECM. Combined, our results support a model where CD8^+^ T cells mediate ECM via direct recognition of cognate antigen on target cells without the need for additional *in situ* secondary activation in the brain by professional APCs and without causing apoptosis.

## Results

### Parasitaemia, neurological symptoms and cerebral vascular leakage during infection with *Pb* ANKA

**Fig 1:**
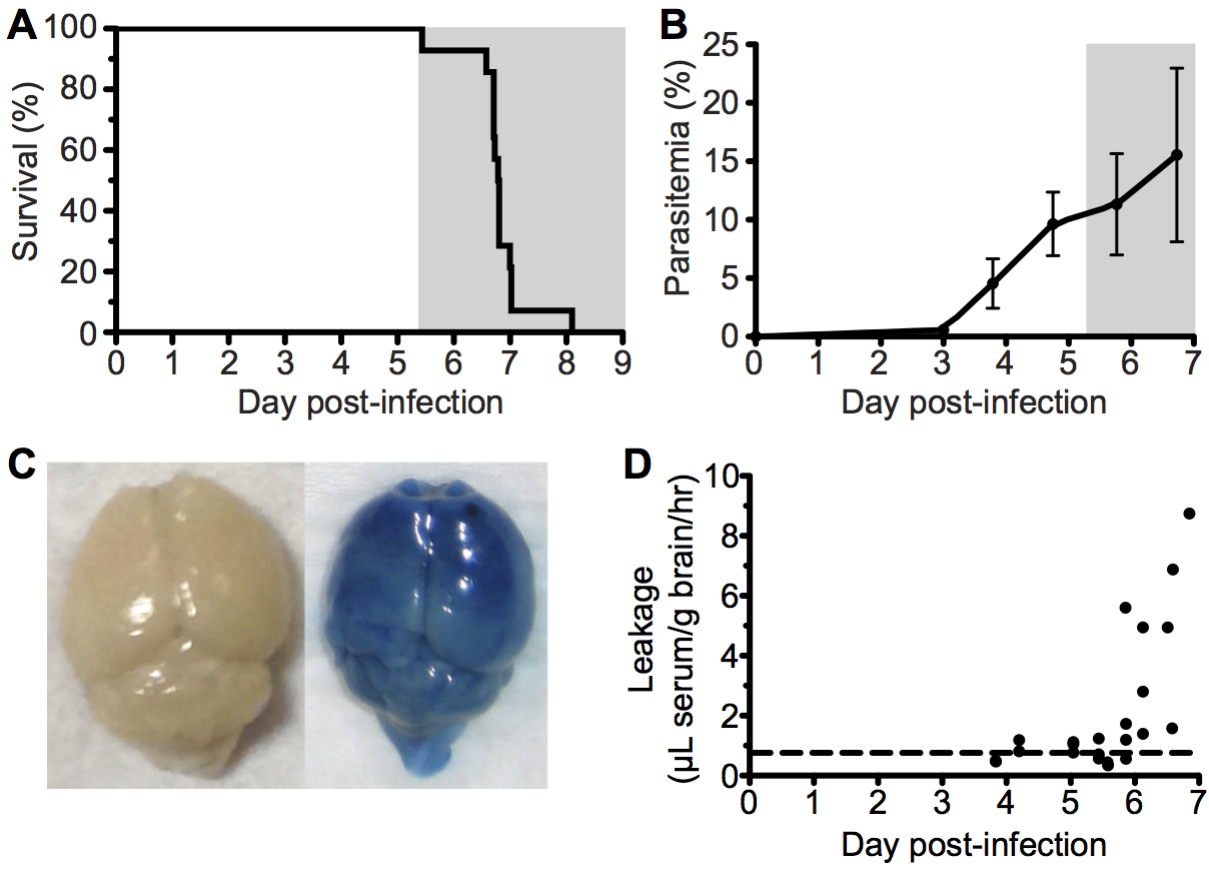
ECM with associated late stage vascular leakage. C57BL/6 (n = 14) mice were intravenously infected with 10^6^ *Pb* ANKA-pRBCs. **(A)** Survival and **(B)** peripheral parasitemia ± SD were monitored daily during development of ECM (grey area). (n = 14, pooled from 2 experiments). **(C)** Representative example of Evans blue leakage in the brain of an uninfected mouse and a mouse with ECM (day 6 p.i). **(D)** Quantification by spectrophotometry of Evans blue leakage in the brains of infected mice on days 4–7 p.i. Dashed line indicates baseline Evans blue signal (no leakage) from uninfected brains. (n = 23, pooled from 4 experiments).

To investigate the immunopathological events that contribute to the development of ECM, we used the well-characterized *Pb* ANKA infection of C57BL/6 mice. Infected mice developed fatal neurological symptoms of ECM on day 6–7 post infection (p.i.) **(Fig 1A),** with a peak peripheral parasitaemia of around 15% **(Fig 1B)**. The brains of symptomatic mice (day 6 p.i) displayed extensive vascular leakage, as assessed by Evans blue leakage, with diffuse blue coloration throughout the brain along with a few intense blue foci, which identify sites of petechial hemorrhage **(Fig 1C)**. In contrast, brains from uninfected mice showed no discoloration **(Fig 1C)**. Spectrophotometric quantification of Evans blue extravasation due to disruption of the cerebral vascular integrity revealed this to be a late occurring phenomenon, coinciding with the onset of ECM. **(Fig 1D)**.

### Cytoadherance of pRBCs in brain during malaria infections

**Fig 2:**
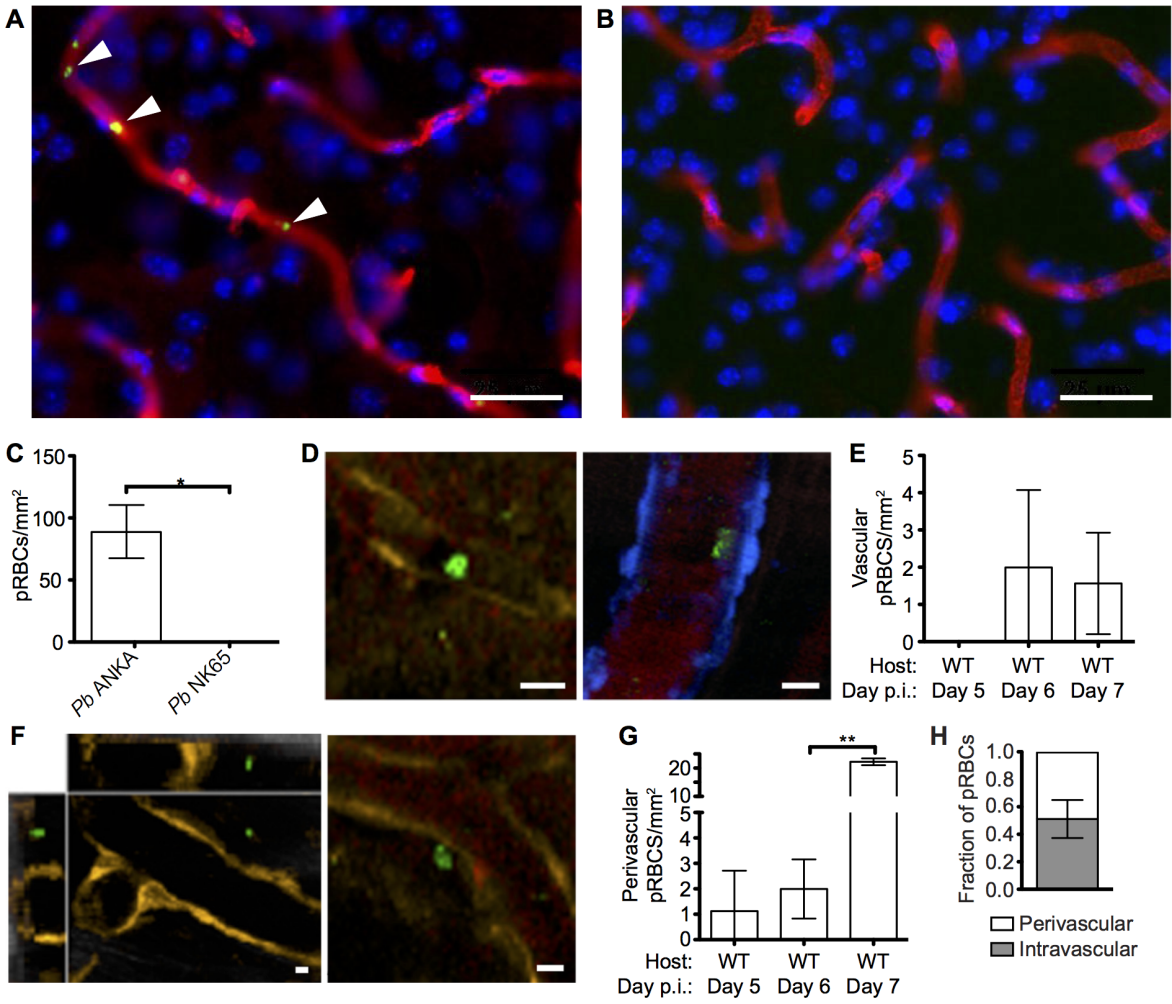
pRBCs make transient adhesive contact with endothelial cells and are deposited within the perivascular space of the meninges of mice with ECM. C57BL/6 mice were infected with 10^4^ *Pb* ANKA-GFP (n = 3) or *Pb* NK65-GFP (n = 3) pRBCs. Representative images demonstrating the **(A)** presence and **(B)** absence of cytoadherent GFP^+^ pRBCs (green) within the cortical vasculature (red) of brains taken on d7 p.i. from transcardially perfused mice infected with *Pb* ANKA or *Pb* NK65 respectively. Cell nuclei are shown in blue. **(C)** Quantification of cytoadherent GFP^+^ pRBCs within brains taken from transcardially perfused mice infected with *Pb* ANKA or *Pb* NK65. CFP and DsRed mice showing blue and orange endothelial cells, respectively, were infected with 10^6^ *Pb* ANKA-GFP pRBCs and monitored for symptoms of ECM. Transcranial two-photon microscopy of the meninges was performed on days 5 (n = 2), 6 (n = 7) and 7 p.i. (n = 3). Circulatory blood flow was visualized by intravenous injection of Evans blue (red) prior to two-photon imaging. **(D)** Example of rare GFP^+^ pRBCs (green) in contact with the luminal side of endothelial cells (orange, left panel or blue, right panel) of DsRed and CFP mice with ECM. **(E)** Quantification of adherent intraluminal pRBCs in cortical pial microvessels on days 5, 6 and 7 p.i. **(F)** Orthogonal (left) and maximum intensity projection (right) examples of GFP^+^ pRBCs (green) located within the perivascular space surrounding the pial microvessels (orange) of a DsRed mouse with ECM. **(G)** Quantification of pRBCs located within the perivascular space surrounding the pial microvessels on days 5, 6 and 7 p.i. with *Pb* ANKA. Endothelial cells are identified by expression of CFP (blue) or DsRed (orange) **(H)** Proportion of pRBCs found either adhering to the luminal vessel wall or located within the perivascular space on day 6 p.i (n = 4). Bars represent mean number ± SD. Scale bars: 5 μm. **p<0.01 (unpaired t test with Welch’s correction).

A pathological hallmark of human CM is sequestration, or cytoadhesion, of parasitized RBCs (pRBCs) within the cerebral blood vessels [35, 36]. In agreement, utilizing static immunofluorescence detection methods, we observed low numbers of *Pb* ANKA pRBCs adhering to vascular endothelial cells in mice with advanced symptoms of ECM **(Fig 2A and 2C)**. Importantly, no parasite accumulation was observed in the brains of mice during *Pb* NK65 infection **(Fig 2B)**, a strain of malaria that causes similar peripheral parasite burdens but does not cause signs of cerebral dysfunction **(S1 Fig)**. We subsequently performed intravital imaging through the thinned skull to study the nature of pRBC adhesion to vascular endothelial cells under physiological flow conditions during ECM. Comparable with results recently obtained by Nacer *et al*. [13], we observed low frequencies of *Pb* ANKA pRBC adhering to vascular endothelial cells **(Fig 2D and 2E)**. This interaction was weak, and the pRBCs were quickly removed by the sheer force of blood flow **(S1 Video)**. Surprisingly, by intravital imaging we also observed an increase in the number of pRBCs located in the perivascular space in mice with advanced symptoms of ECM **(Fig 2F and 2G, S2 Video)**. In fact, on day 6 p.i., when the majority of mice developed ECM (9/14), perivascular pRBCs (∼3 pRBC/mm^2^) were found more often than adherent luminal pRBCs (∼2 pRBC/mm^2^). In those mice that developed ECM on day 7 p.i., there was a further significant increase in numbers of perivascular pRBCs (∼22 pRBC/mm^2^), which represents a 10-fold increase that is substantially more than that of peripheral parasitaemia (1.5-fold increase), or adherent luminal pRBCs (no increase), observed between days 6 and 7 p.i. In contrast to LCMV encephalitis, another CD8^+^ T cell-dependent immunopathological model, we did not observe any evidence of petechial hemorrhages during intravital imaging of meninges of mice with ECM. Thus, it is unlikely that the accumulation of pRBCs within the perivascular space during ECM was simply due to formation of and carriage within hemorrhages. **(S2 Fig and S3 Video)**. These results show that parasite accumulation within the brain is a specific event associated with ECM. However, at the point of ECM development, perivascularly located pRBCs, determined as such by their location relative to the fluorescent endothelium, are at least as common as briefly adherent intravascular pRBCs **(Fig 2H)**. The interaction of pRBCs or parasite-derived material with cells behind the blood vessel endothelial wall within the perivascular space may, therefore, be an important event in the development of ECM.

### Comparable recruitment and activation of CD8^+^ T cells in the brains of mice infected with ECM-causing and non-ECM-causing *P*. *berghei* infections

**Fig 3:**
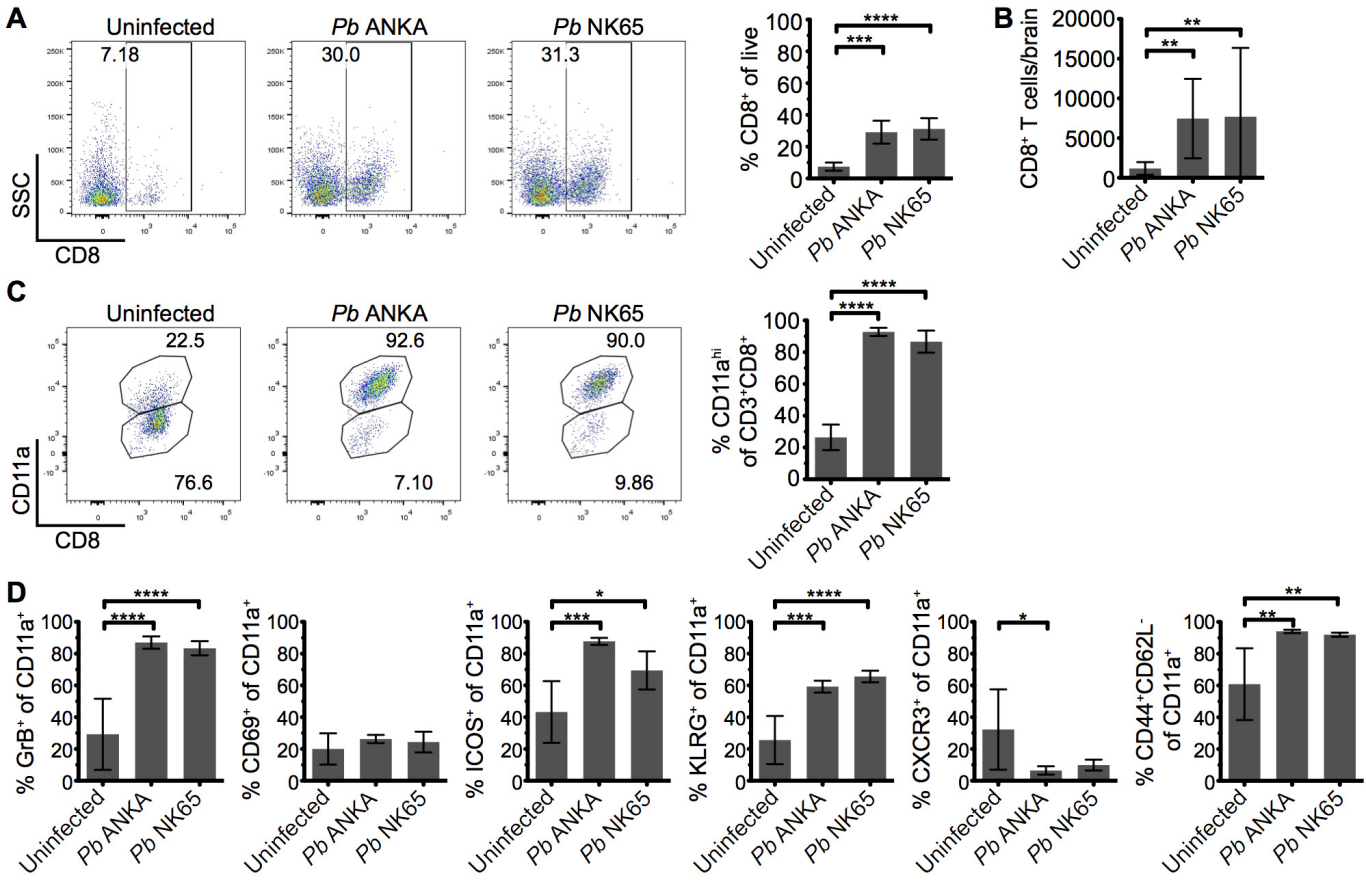
Parasite specific CD8^+^ T cells are comparably activated within the brains of mice infected with *Pb* ANKA and *Pb* NK65 parasites. C57BL/6 mice were infected with 10^4^ *Pb* ANKA or *Pb* NK65 pRBCs. **(A)** Representative flow cytometric plots and calculated percentages showing frequencies of CD8^+^ T cells (gated on live leukocytes) within the brains of uninfected and infected (day 7 p.i.) mice (n = 5). **(B)** Absolute numbers of CD8^+^ T cells within the brains of uninfected and infected (day 7 p.i.) mice (n = 19–22). **(C)** Percentage of CD8^+^ T cells expressing high levels of the surrogate antigen specificity marker, CD11a (n = 5). **(D)** Activation phenotype of CD8^+^CD11a^high^ T cells from uninfected and infected (day 7 p.i.) mice (n = 5). Results are representative of two independent experiments **(A, C and D)** or four combined experiments **(B)**. Bars represent mean number ± SD. *p ≤ 0.05, **p ≤ 0.01, ***p ≤ 0.001, ****p ≤ 0.0001 (one-way ANOVA with Tukey's multiple comparisons test).

CD8^+^ T cells are known to play a central role in the development of ECM [11, 17], yet the mechanisms through which they promote cerebral pathology during malaria infection remains poorly understood. It has been shown that despite the divergent infection outcomes, CD8^+^ T cells accumulate at similar levels in the brains of mice infected with *Pb* ANKA and *Pb* NK65 parasites [37]. We therefore hypothesized that brain accumulating CD8^+^ T cells exhibited intrinsic differences in phenotype activation status during *Pb* ANKA and *Pb* NK65 infections, explaining their pathogenic activity specifically during ECM-inducing infections. To test this hypothesis, we isolated leukocytes from the brains of infected and uninfected mice and characterized them via flow cytometry. As reported, similar frequencies and numbers of CD8^+^ T cells accumulated within the brains of mice infected with *Pb* ANKA and *Pb* NK65 on day 7 p.i., when mice infected with Pb ANKA developed ECM **(Fig 3A and 3B)**. Moreover, comparable frequencies of brain accumulating CD8^+^ T cells expressed high levels of CD11a during *Pb* ANKA and *Pb* NK65 infections **(Fig 3C)**, a marker of antigen experience [38, 39]. The intracerebral CD8^+^CD11a^high^ parasite-specific T cells also displayed comparable activation in *Pb* ANKA and *Pb* NK65 infections, as evidenced by effector status (CD44^+^CD62L^-^) and increased expression of CD69, ICOS, KLRG1, CXCR3, and granzyme B **(Fig 3D)**. Combined, these results show that CD8^+^ T cells recruited to the brains of mice infected with either ECM-causing or non-ECM causing parasites are similarly activated to cause cerebral pathology and that, although necessary, their presence alone is not sufficient for ECM development.

### T cells are perivascularly located within the brain but display different behaviors in *Pb* ANKA and *Pb* NK65 infected mice

**Fig 4:**
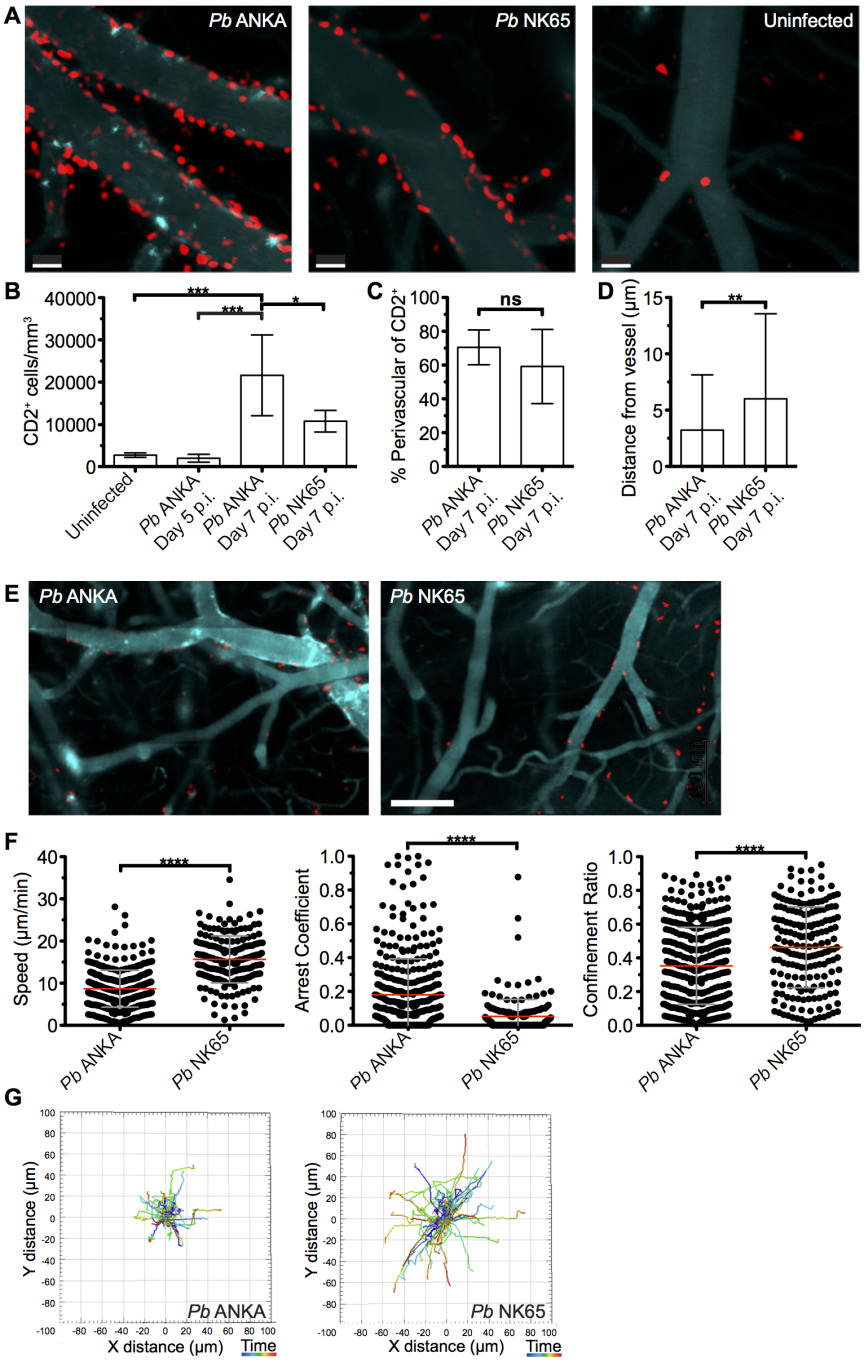
T cells exhibit equivalent perivascular compartmentalisation but distinct behaviours during *Pb* ANKA and *Pb* NK65 infections. hCD2-DsRed C57BL/6 mice were infected with 10^4^ *Pb* ANKA or *Pb* NK65 pRBCs or left uninfected. Transcranial two-photon microscopy of the meninges was performed on days 5 p.i. (*Pb* ANKA), and 7 p.i. (*Pb* ANKA and *Pb* NK65). **(A)** Maximum intensity projections from intravital two-photon microscopy movies (283x283x30 μm) showing Qtracker non-targeted quantum dot-labeled blood vessels (cyan) and DsRed T cells (red) within the meninges of infected mice on day 7 p.i. with *Pb* ANKA and *Pb* NK65 and in uninfected mice. **(B)** Mean number ± SD of hCD2-DsRed T cells (including luminal and perivascular) in imaged tissue sites in uninfected control mice and on day 5 (*Pb* ANKA, n = 2) and 7 p.i. (*Pb* ANKA, n = 7; and *Pb* NK65, n = 5). **(C)** Mean proportion ± SD of hCD2-DsRed T cells located perivascularly in the meninges of *Pb* ANKA and *Pb* NK65 infected (day 7 p.i.) mice. **(D)** Mean distance ± SD (μm) of perivascularly located T cells from abluminal vessel wall in mice infected with *Pb* ANKA and *Pb* NK65 (day 7 p.i.). **(E)** Representative cropped tile scanned images showing heterogeneity of T cell clusters around pial vessels within the brains of mice infected with *Pb* ANKA and *Pb* NK65 (day 7 p.i.). **(F)** Quantification of average perivascular T cell speeds, arrest coefficient (proportion of time points when instantaneous velocity is <2 μm/min) and confinement ratio (track displacement/track length) from individual three-dimensional T cell tracks. Each point represents an individual DsRed T cell. Results are pooled two experiments, n = 4 for both groups. **(G)** Graphical illustrations summarizing the XY movement of individual perivascularly located T cells from normalized starting positions in the brains of mice on day 7 p.i. with *Pb* ANKA and *Pb* NK65 parasites. Scale bars: (A) 30 μm, (C) 150 μm. *p ≤ 0.05, ****p<0.0001 (Student’s unpaired t test or one-way ANOVA with Tukey's multiple comparisons test).

The comparable activation status of CD8^+^CD11a^high^ parasite specific T cells in the brains of mice infected with *Pb* ANKA and *Pb* NK65 parasites on day 7 p.i. suggested that the disparate pathogenic activity of the cells during the two infections may be driven by CD8^+^ T cell extrinsic factors, which were not quantifiable through traditional flow cytometric analysis. To investigate this, we performed intravital imaging to study the compartmentalization and dynamics of T cells within the brain of hCD2-DsRed transgenic B6 mice during *Pb* ANKA and *Pb* NK65 infections. Few DsRed^+^ T cells were identified within the brains of naïve mice **(Fig 4A)** or on day 5 p.i. with *Pb* ANKA **(S3 Fig)**, confirming the late accumulation of T cells in the brain during infection. Very few NK cells or B cells, which may express hCD2, were observed in the brains of infected mice on day 7 p.i. **(S4 Fig)**. Characterization of T cells from isolated meningeal vessels of infected mice on day 7 p.i. showed them to be mainly CD8^+^ (>70%) **(S5 Fig)**. The number of T cells/mm^2^ of vessel was increased within the brains of *Pb* ANKA and *Pb* NK65 infected mice (day 7 p.i.) compared with uninfected mice **(Fig 4A and 4B)**. The relatively high numbers of T cells quantified by two photon microscopy compared with those given by whole brain flow cytometric analysis may reflect preferential meningeal T cell localization, diluted out by whole brain homogenization. Alternatively it may represent the failure to recover for flow cytometric analysis the *Pb* specific T cells that are tightly bound to target cells during ECM, a problem recently highlighted for T cell isolation from non-lymphoid tissues [40].

Surprisingly, the majority of T cells were compartmentalized to the perivascular side of the blood vessels during both *Pb* ANKA and *Pb* NK65 infections (70.5±10.3% vs 59.2±21.9%, respectively) **(Fig 4C)**. Perivascularly located T cells were closely associated with the abluminal surface of blood vessels in mice infected with *Pb* ANKA and *Pb* NK65 parasites. Average distance from the vessel surface was, however, greater in mice infected with *Pb* NK65 than *Pb* ANKA parasites (5.9±7.4 μm v 3.1±4.8 μm, respectively) **(Fig 4D)**. Interestingly, the distribution of T cells was not homogenous around the vessels imaged, with clusters of T cells observed preferentially around particular vessels **(Fig 4E)**, which are most likely post-capillary venules [20].

It is becoming evident that the behavior of effector T cells, such as interaction with antigen presenting cells or responsiveness to chemokines and adhesion molecules, determines the local activity and function of the cells within the tissue [41, 42]. Thus, as T cells, of which greater than 70% are CD8^+^, were observed in comparable compartments of the brain during *Pb* ANKA and *Pb* NK65 infections, we next examined whether the cells displayed disparate behavior in the two infections, underlying their pathogenic activity specifically during *Pb* ANKA infection. Tracking DsRed^+^ cells revealed that perivascular T cells within brains of mice infected with *Pb* ANKA **(S4 Video and S5 Video)** exhibited a more arrested phenotype than those in mice infected with *Pb* NK65 **(S6 Video and S7 Video)**. These differences were reflected in a lower mean track speed (8.7±4.4 v 15.7±5.6 μm/min), higher arrest coefficient (0.18±0.21 v 0.05±0.1) and lower confinement ratio (0.35±0.23 v 0.46±0.24), reflecting greater confinement **(Fig 4F)**. The more constrained movement of perivascular T cells in brains of mice infected with *Pb* ANKA compared with *Pb* NK65 parasites is graphically illustrated by plotting 2D projections of tracks over 17 minutes that were then fixed to a common origin (**Fig 4G**). Thus, a major correlate of ECM is the perivascular arrest of CD8^+^ T cells.

### Perivascular T cells within the subarachnoid space of mice with ECM form stable interactions with CX_3_CR1^+/GFP^ cells

**Fig 5:**
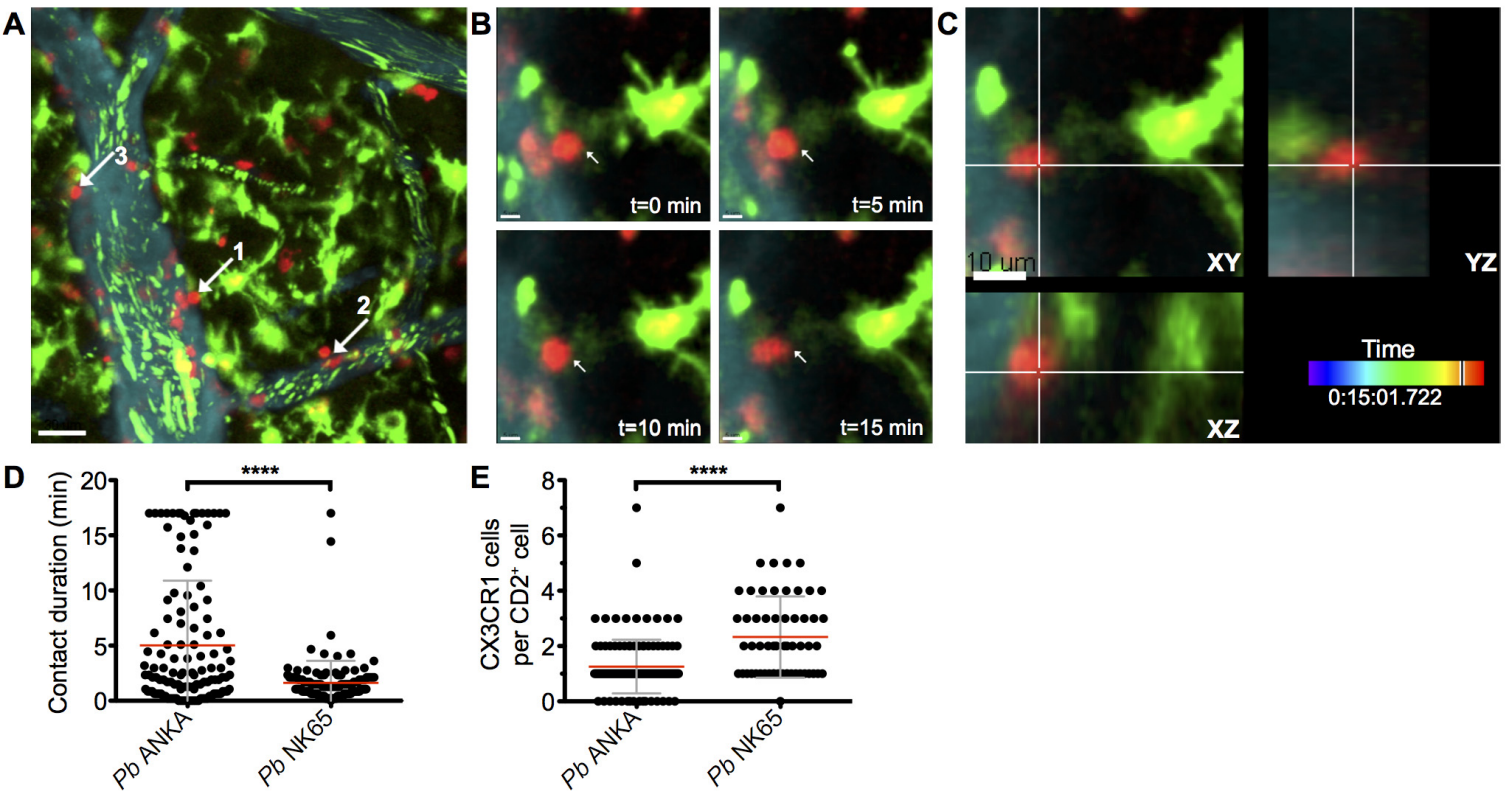
Perivascular T cells form long-lasting interactions with CX_3_CR1^+/GFP^ in the brains of mice infected with *Pb* ANKA. hCD2-DsRed X CX_3_CR1^GFP/GFP^ dual reporter mice were infected with 10^4^ *Pb* GFP ANKA (n = 5 from three experiments). **(A)** Maximum intensity projections from intravital two-photon microscopy movies (283x283x30 μm) showing interaction of perivascular T cells (red) with CX_3_CR1^+/GFP^ cells (green) in brains of mice on day 7 p.i. Blood vessels (cyan) were visualized by i.v. injection of Qtracker non-targeted quantum dots. GFP^+^ pRBCs (green) can also be seen within the lumen of the vessels. White arrows highlight selected perivascular T cells forming stable interactions with CX_3_CR1^+/GFP^ cells. **(B)** Selected cropped frames from a time-lapse movie showing a perivascular T cell [number one in **(A)** in contact with a CX_3_CR1^+/GFP^ cell over a 17-minute period. **(C)** Three dimensional section showing the same T cell and CX_3_CR1^+/GFP^ cell interacting in the XY, XZ and YZ planes. Scale bars: (A) 30 μm, (B) 5 μm, (C) 10 μm. (D) Mean duration ± SD of individual hCD2-DsRed T cell contacts with CX_3_CR1^+/GFP^ cells in **S8 and S9 Videos**. (E) Mean number ± SD of CX_3_CR1^+/GFP^ cells contacted by individual perivascular hCD2-DsRed T cells in **S8 and S9 Videos**, over a 17-minute period.

The arrest of perivascular T cells within brains of mice infected with *Pb* ANKA was consistent with immune synapse formation with antigen-expressing cells. The interaction of perivascular T cells with brain APCs has previously been shown to instruct T cell pathogenic functions in other models of T cell mediated cerebral pathology [43, 44]. Consequently, we next assessed the interaction of T cells with professional APC populations in the brains of mice infected with *Pb* ANKA and *Pb* NK65 utilizing hCD2-DsRed X CX_3_CR1^+/GFP^ dual reporter mice, where GFP is expressed by subsets of monocytes, macrophages and DCs, and all microglia [45]. We found that perivascular T cells within brains of mice infected with either *Pb* ANKA or *Pb* NK65 were closely associated with CX_3_CR1^+/GFP^ cells and made frequent interactions with their cellular processes **(S8 Video and S9 Video)**. Consistent with results from the single-reporter hCD2-DsRed mice, arrested perivascular T cells were more numerous in mice infected with *Pb* ANKA than *Pb* NK65, and many were stably bound to CX_3_CR1^+/GFP^ cells **(Fig 5A–5C, S10 Video)**. Formation of longer lasting stable interactions between CX_3_CR1^+/GFP^ cells and perivascular T cells was reflected by longer average contact times for individual perivascular T cells **(Fig 5D)** and the formation of fewer contacts with new CX_3_CR1^+/GFP^ cells in mice infected with *Pb* ANKA than in mice infected with *Pb* NK65 **(Fig 5E)** over the course of the 17 minute imaging period. Combined with CD8^+^ T cell recruitment results, this raised the possibility that differences within the CX_3_CR1^+/GFP^ population may determine the behavior, and associated pathogenicity, of perivascularly located T cells that enter the brain subsequent to their full activation in the spleen during *Pb* infection.

### CX_3_CR1^+/GFP^ cells with an activated phenotype accumulate within the brain during infection with ECM-causing and non-ECM-causing *P*. *berghei* infections

**Fig 6:**
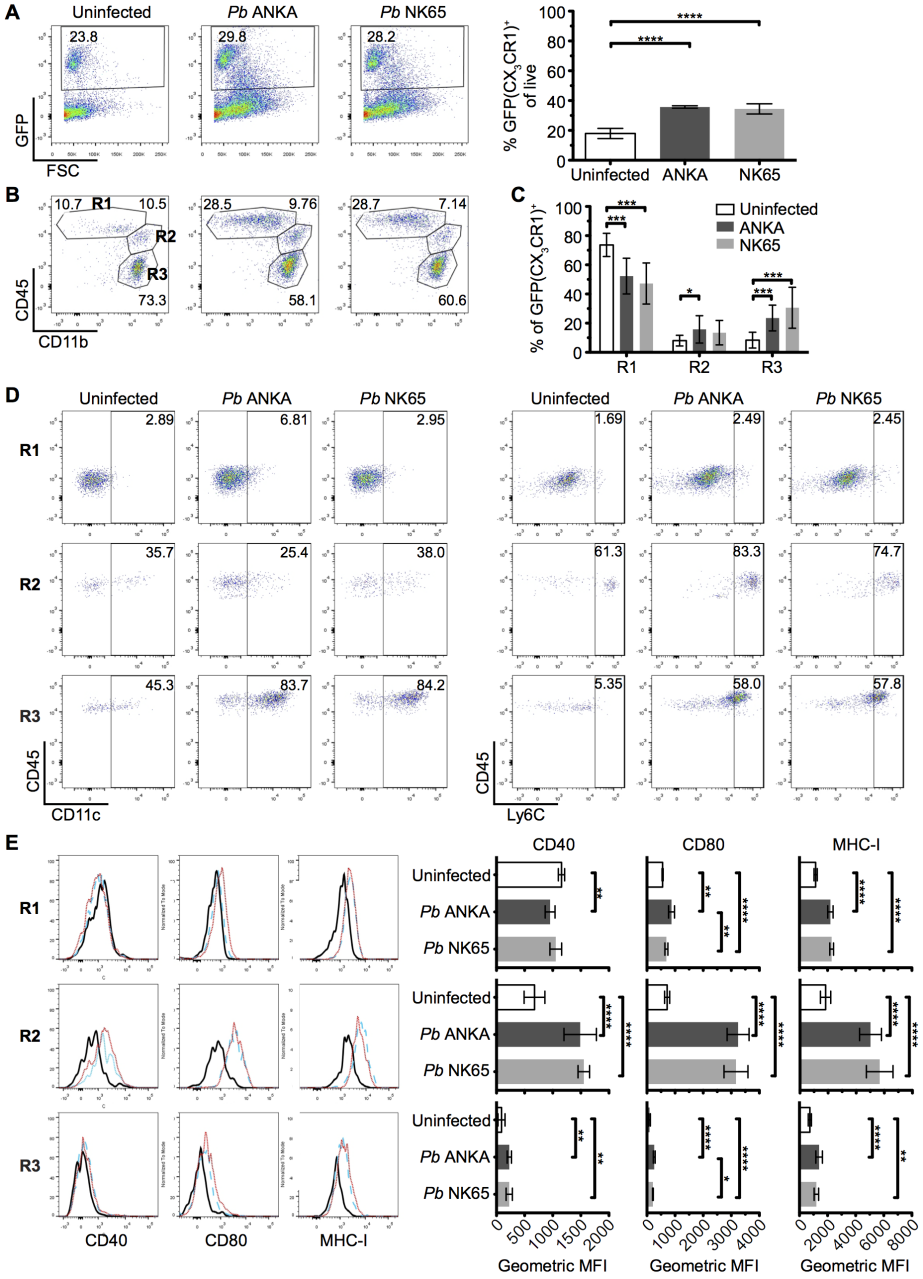
The intracerebral CX_3_CR1^+/GFP^ cellular response is comparable during *Pb* ANKA and *Pb* NK65 infections. CX_3_CR1^+/GFP^ mice were infected with 10^4^ *Pb* ANKA or *Pb* NK65 pRBCs. Brains from uninfected and infected (day 7 p.i.) mice were analyzed by flow cytometry. **(A)** Representative plots and calculated percentages of CX_3_CR1^+/GFP^ cells (gating on live leukocytes) within the brains of uninfected and infected (day 7 p.i.) mice (n = 6). **(B)** Representative plots showing the subdivision of GFP^+^ cells into: R1—CD45^int^CD11b^hi^ microglia; R2—CD45^hi^CD11b^hi^ meningeal, perivascular macrophages and inflammatory monocytes; R3—CD45^hi^CD11b^int^ leukocytes. **(C)** Calculated percentages of R1, R2 and R3 sub-gated populations within the brains of uninfected and infected (day 7 p.i.) mice (gating on live GFP^+^ leukocytes) (n = 15–17). **(D)** Representative dot plots showing the expression of phenotypic markers CD11c and Ly6C on the gated R1, R2 and R3 GFP^+^ populations from brains of uninfected and infected (day 7 p.i.) mice. **(E)** Representative histograms and calculated geometric means of CD40, CD80 and MHC I expression on the gated R1, R2 and R3 GFP^+^ populations from brains of uninfected and infected (day 7 p.i.) mice (n = 6). Results are representative of at least two independent experiments **(A, B, D and E)** or are pooled from three combined experiments **(C)**. Bars represent mean number ± SD. *p ≤ 0.05, **p ≤ 0.01, ***p ≤ 0.001, ****p ≤ 0.0001 (one-way ANOVA with Tukey's multiple comparisons test).

We hypothesized that the different nature of cognate interaction between T cells and CX_3_CR1^+/GFP^ cells in the brains during *Pb* ANKA and *Pb* NK65 infections was due to alterations in the composition and/or activation of the brain CX_3_CR1^+/GFP^ population during infection with *Pb* ANKA and *Pb* NK65. Thus, to more specifically identify and characterize the CX_3_CR1^+/GFP^ cells present within the brain during the two infections, we isolated GFP^+^ leukocytes from the brains of infected and uninfected mice and characterized them for expression of phenotypic and functional markers. The frequencies of GFP^+^ cells (out of total leukocytes) in the brain increased comparably during infection with both *Pb* ANKA and *Pb* NK65 **(Fig 6A)**. We subsequently sub-gated GFP^+^ cells into three subsets based on expression of CD11b and CD45; R1 = CD45^int^CD11b^hi^ microglia, R2 = CD45^hi^CD11b^hi^ meningeal and perivascular macrophages and inflammatory monocytes and R3 = a mixed population of CD45^hi^CD11b^int^ leukocytes [46, 47] **(Fig 6B)**. The majority of GFP^+^ cells in brains from uninfected mice were microglial cells (73.7±7.9%). During both infections, the proportion of microglia within the GFP^+^ population decreased (52.3±12.2% for *Pb* ANKA, 47.2±14.1% for *Pb* NK65), likely due to other GFP^+^ cells infiltrating the brain **(Fig 6C)**. CD45^hi^CD11b^int^ leukocytes were mainly CD11c^+^ DCs (*Pb* ANKA 82.8±10.3%, *Pb* NK65 74.5±8.6%) and CD45^hi^CD11b^hi^ leukocytes were mainly Ly6C^hi^ inflammatory monocytes (*Pb* ANKA 84.4±2.5%, *Pb* NK65 83.8±2.5%). Lack of CD11c and Ly6C expression on the CD45^int^ CD11b^hi^ population confirmed their identity as microglia **(Fig 6D)**. These results demonstrate that the composition of the CX_3_CR1^+/GFP^ population changed within the brain during malaria infection, and that it changed comparably during ECM-causing and non-ECM-causing malaria infection. Furthermore, in contrast to our hypothesis, infection with both *Pb* ANKA and *Pb* NK65 caused largely comparable activation of all three CX_3_CR1^+/GFP^ populations with the most striking up-regulation of co-stimulatory (CD40 and CD80) and antigen presenting molecules (MHC-I) occurring in the CD45^hi^CD11b^hi^ population **(Fig 6E)**. Changes in T cell motility correlating with ECM could not be attributed to different T cell or myeloid cell surface phenotypes and, thus, *in vivo* motility represents a distinct parameter of value in assessing T cell function.

### CX_3_CR1^+^ cells are dispensable for induction of ECM

**Fig 7:**
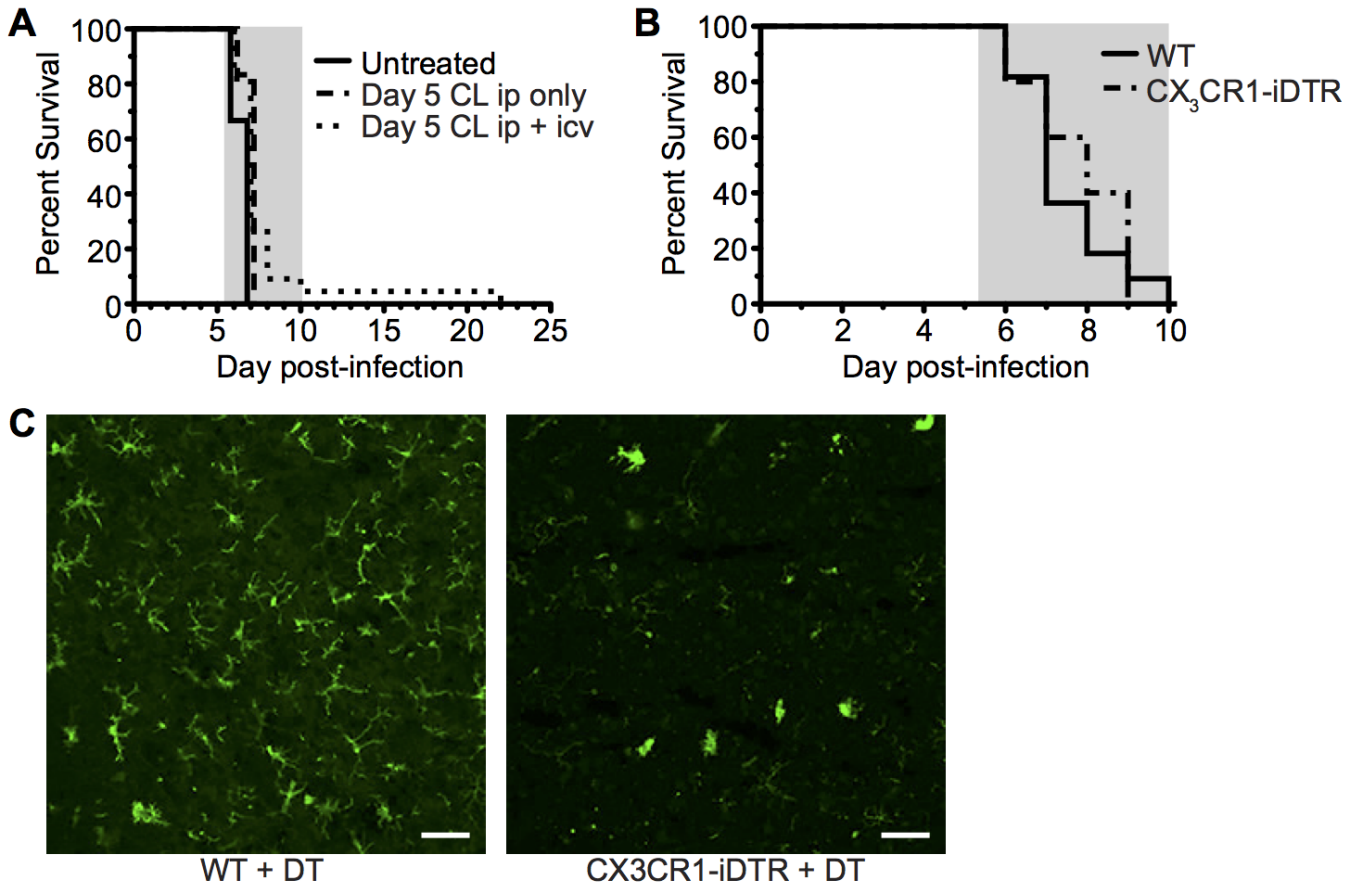
Depletion of systemically and perivascularly located phagocytic cells from day 5 p.i. does not prevent ECM. **(A)** C57BL/6 mice were infected with 10^4^ *Pb* ANKA pRBCs. On day 5 p.i. mice were injected i.p. with 300 μL clodronate liposomes with (n = 22) or without (n = 6) 8 μL clodronate liposomes i.c.v., or left untreated (n = 9). Mice were monitored daily for development of ECM (grey area). Results are pooled from five experiments. **(B)** C57BL/6 (n = 11) and CX_3_CR1-iDTR mice (n = 5) were infected with 10^6^ *Pb*ANKA pRBCs and treated with tamoxifen and diphtheria toxin as described in the methods to systemically deplete CX_3_CR1^+^ cells in the latter group. Mantel-Cox test showed no statistically significant differences in survival. **(C)** Representative coronal cryosection of brains showing depletion of Iba1^+^microglia (green), a CX_3_CR1^+^ population, in CX_3_CR1-iDTR mice (right), compared to parallel C57BL/6 control (left), with ECM. Scale bars: (C) 50 μm.

The CD45^hi^CD11b^hi^ monocyte and macrophage containing population (R2) displayed the most activated phenotype during infection and were also found to be enriched within the meninges, the site of imaging, compared with the whole brain **(S6 Fig)**. Previous studies investigating the role of monocytes and macrophages in ECM pathogenesis have employed systemic administration of clodronate liposomes, CCR2 and Gr1 depleting antibody or AP20187 drug administration to MAFIA mice [19, 26, 48, 49]. None of these interventions prevented ECM development when given late in the course of infection. Crucially, however, these methods did not deplete populations located behind the endothelial barrier, including meningeal and perivascular macrophages or microglia. Accordingly, we addressed the contribution of these cells, and by association the importance of T cell interactions with them, in the development of ECM. Depletion of systemic and meningeal and perivascular macrophages through combined intraperitoneal (i.p.) and intracerebroventricular (i.c.v.) injection of clodronate liposomes (C.L.) using a protocol adapted from Galea *et al*. [50], from day 5 p.i. failed to protect mice against development of ECM **(Fig 7A)**. Furthermore, specific depletion of CX_3_CR1 expressing cells, including perivascular and meningeal macrophages and microglia, from day 3 of infection utilizing CX_3_CR1-iDTR mice [51] also failed to inhibit development of ECM **(Fig 7B)**. This was despite large scale depletion of brain resident macrophages and microglia, as shown by reduced Iba1 staining **(Fig 7C)**. Our results, therefore, indicate that although stable interaction of T cells with CX_3_CR1^+/GFP^ APCs is a frequent event, specifically during *Pb* ANKA infection, this activity is redundant for the development of ECM.

### Parasite-specific CD8^+^ T cells recapitulate ECM pathology in resistant TCR Tg mice

**Fig 8:**
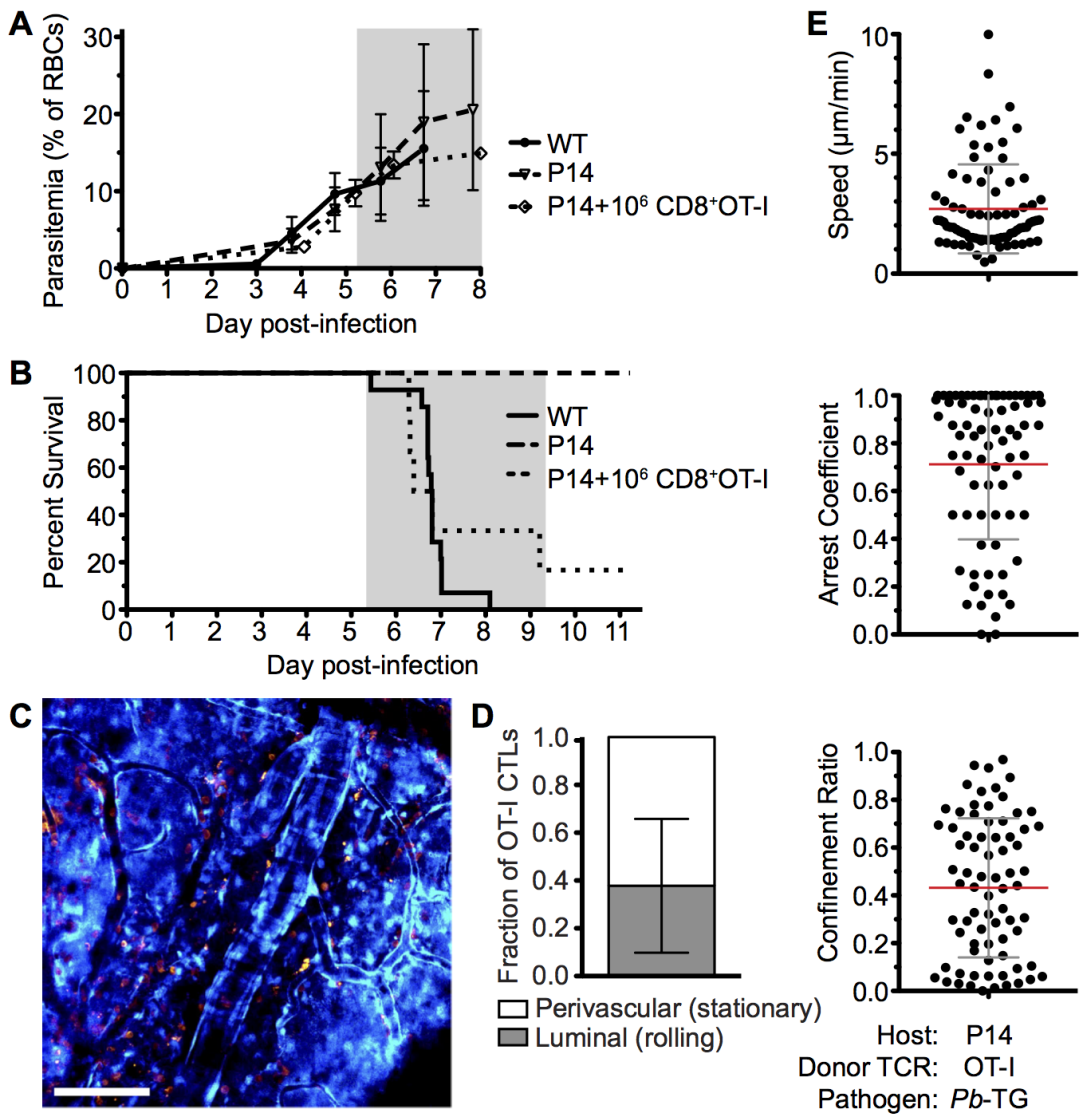
Parasite specific OT-I CD8^+^ T cells that directly cause ECM are perivascular and are highly arrested in the brain. C57BL/6 (n = 9) and P14 (n = 10) mice were infected with 10^6^ SIINFEKL-expressing *Pb*-TG pRBCs. Prior to infection, 10^6^ naïve DsRed-expressing OT-I CD8^+^ T cells were adoptively transferred into CFP^+^ P14 host mice (n = 6). Development of ECM (grey area) was monitored by assessing **(A)** peripheral parasitaemia ± SD and **(B)** survival. **(C)** Maximum intensity projections from an intravital two-photon microscopy movie showing accumulation of DsRed^+^CD8^+^ OT-I T cells (orange) within the brain of a P14 recipient on day 6 p.i. infection. Endothelial cells (blue) were visualized by CFP expression. **(D)** Proportion ± SD of CD8^+^ OT-I T cells located perivascularly on day 6 p.i. (n = 8). **(E)** Quantification of average perivascular T cell speeds, arrest coefficient and confinement ratio from individual three-dimensional T cell tracks. Results are pooled from two experiments.

To further analyze the mechanisms through which CD8^+^ T cells mediate ECM, we developed a tractable Ag-specific model, where CD8^+^ T cells with bona fide pathogenic activity can be visualized and tracked. Adoptive transfer of 10^6^ SIINFEKL-specific OT-I CD8^+^ T cells into otherwise ECM-resistant P14 TCR transgenic mice, in which most CD8^+^ T cells express the receptor specific for the gp33 epitope of LCMV [52], led to the robust development of ECM when mice were subsequently infected with a GFP-SIINFEKL-expressing strain of *Pb* ANKA parasite (*Pb*-TG) **(Fig 8A and 8B)**. Consistent with results obtained when examining the polyclonal T cell response **(Fig 4)**, DsRed-expressing OT-I CD8^+^ T cells were recruited to the brain of P14 hosts in response to infection with *Pb*-TG (**Fig 8C**). These brain recruited OT-I CD8^+^ T cells were predominantly perivascular, as determined by their location relative to the fluorescent endothelium (**Fig 8D**). Perivascular OT-I CD8^+^ T cells were, however, more highly arrested than the polyclonal T cells with a mean speed of 2.36±2.32 μm/min and a mean arrest coefficient of 0.829±0.256 **(Fig 8E, S11 Video)**. Differences in Ag-specific and polyclonal T cell behavior are likely due to the varied specificity of the complex polyclonal CD8^+^ T cell population for different parasite molecules, heterogeneously expressed by cells within the brain, [53]. Transferred OT-I CD8^+^ T cells behaved comparably in infected wild type and P14 hosts, indicating that their highly arrested behavior was not an artifact of the transgenic recipient **(S7 Fig)**. As our results argue against the requirement for secondary activation by brain APCs to endow brain-infiltrating CD8^+^ T cells with pathogenic activity during *Pb* ANKA infection **(Fig 3)**, it is likely that the arrested T cells are attached directly to antigen-presenting target cells, and that such interaction contributes to ECM pathogenesis. These results further emphasize that perivascular arrest of antigen-specific CD8^+^ T cells is a consistent signature of ECM.

### ECM induces increased apoptosis of brain endothelial cells but not sufficiently to account for widespread vascular leakage

**Fig 9:**
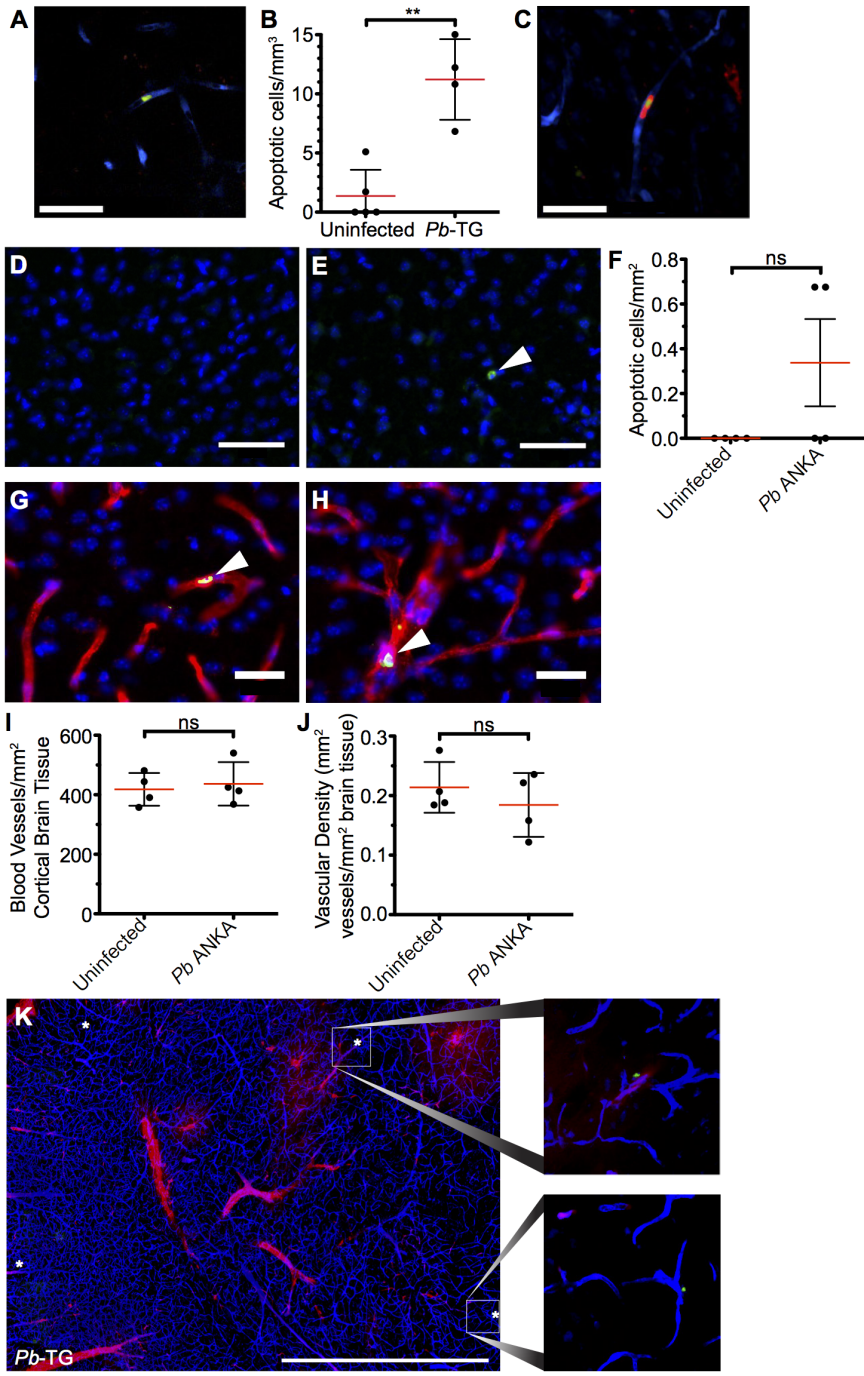
Widespread apoptosis or loss of vasculature does not occur in the brains of mice with ECM. **(A-C and K)** 10^6^ DsRed^+^CD8^+^ OT-I T cells were adoptively transferred into CFP^+^ P14 host mice, which were subsequently infected with 10^6^ SIINFEKL-expressing *Pb*-TG pRBCs (n = 4) or left uninfected (n = 5). Mice were intravenously injected with CellEvent Caspase-3/7 Detection Reagent when displaying signs of ECM (day 6 p.i). Brains were subsequently isolated and processed for histological examination. **(A)** Representative snapshot of an apoptotic cell (green) associated with the cortical vasculature (blue) of the brain taken from a mouse with ECM. **(B)** Quantification ± SD of apoptotic cells stained with CellEvent per mm^3^ in brains from infected and uninfected control mice. **(C)** Representative snapshot showing an apoptotic cell (green) in contact with a parasite-specific CD8^+^ OT-I T cell (red). Endothelial cells (blue) were visualized by CFP expression. **(D-J)** C57BL/6 mice were infected with 10^4^ *Pb* ANKA pRBCs or left uninfected. Brains were removed and processed for histological examination when *Pb* ANKA mice developed signs of ECM (day 7 p.i.). Representative images demonstrating the **(D)** absence and **(E)** rarity of apoptotic cells (green) within the cortex of brains from uninfected and ECM affected mice respectively. Cell nuclei are shown in blue. **(F)** Quantification ± SD of apoptotic cells stained for activated caspase 3 per mm^2^ in brains from infected and uninfected control mice. **(G-H)** Co-staining of activated caspase 3 (green) and CD31 (red) demonstrating an **(G)** apoptotic endothelial cell and an **(H)** apoptotic leukocyte within the cortical vasculature of brains taken from ECM affected mice. Cell nuclei are shown in blue. Quantification ± SD of total **(I)** number and **(J)** area per mm^2^ of cortical blood vessels in the brain from uninfected and ECM affected mice. **(K)** Representative tile scan showing the localization of CellEvent^+^ apoptotic cells (green/asterisk) in relation to vascular leakage (red) within the brain of an ECM affected (day 6 p.i.) CFP^+^ P14 mouse. Endothelial cells (blue) were identified by CFP. Scale bars: (A,C) 25 μm, (D,E) 50 μm, (G,H) 25 μm, (L) 1 mm. Results are pooled from mice infected in one experiment. Significance determined by unpaired t test with Welch’s correction.

It has recently been proposed that CD8^+^ T cells can directly interact with antigen expressing endothelial cells, and that this interaction may be a proximal event in causing endothelial cell apoptosis and ECM development [24, 26]. We, therefore, assessed the level of apoptosis within the brains of mice with ECM. Detection of apoptotic cells during intravital imaging was attempted by i.v. injection of an active-caspase3/7-reporter molecule, CellEvent. However, no CellEvent-positive cells were observed by transcranial microscopy, suggesting that cellular apoptosis is a rare event in the brain during ECM. This was confirmed by *ex vivo* examination of thick coronal sections from mice injected with CellEvent, allowing for a larger volume of the brain to be surveyed **(Fig 9A)**, with only 11.2 cells/mm^3^ apoptotic cells found in the vasculature of the cortex of brains of mice with ECM, compared with 1.36 cells/mm^3^ in naïve brains **(Fig 9B)**. Although rare, apoptotic cells could be found in immediate contact with parasite-specific CD8^+^ T cells **(Fig 9C)**, suggesting that the CD8^+^ T cells were mediating the cellular apoptosis. The low level of cellular apoptosis in the brains of mice with ECM was further confirmed by immunofluorescent staining of thick cortical sections with antibodies against activated caspase 3 **(Fig 9D–9F)**. Brains from mice subject to middle cerebral artery occlusion induced stroke were used to confirm successful activated caspase 3 staining **(S8 Fig)**. Co-staining of activated caspase 3 and CD31 demonstrated that the rare apoptotic cells present during ECM were associated with the vasculature. By examination of morphology, apoptotic cells were identified as both endothelial cells **(Fig 9G)** and vasculature associated leukocytes **(Fig 9H)**. Apoptotic cells may be rapidly cleared, particularly under physiological blood flow conditions, potentially explaining the low levels of apoptosis observed in the brains from mice with ECM. However, no differences were observed in either total number of vessels **(Fig 9I and S9 Fig)** or vessel area **(Fig 9J and S9 Fig)** in brains from mice with ECM compared with uninfected mice. Despite this, as previously reported, extensive vascular leakage was observed in ECM **(Fig 9K)**. Thus, our results indicate that CD8^+^ T cells do not cause extensive vascular leakage via endothelial cell apoptosis; rather, our results support an alternative model in which perforin and granzyme B release, by perivascular parasite-specific CD8^+^ T cells, induces opening of intercellular junctions of the endothelium.

## Discussion

In this study, we have shown that CD8^+^ T cells accumulate at high levels in the perivascular space of the brain during malaria infection. The perivascular compartment of the brain is a hitherto understudied location in the study of ECM pathogenesis, even though, consistent with our results, leukocytes have previously been observed in this space during ECM using electron microscopy [54, 55]. Although, our intravital studies examined the superficial regions of the brain, perivascularly located CD8^+^ T cells were also observed around vessels deeper in the brain during ECM (**S10 Fig**), suggesting that this phenomenon is not unique to pial vasculature. Our results extend the study of Nacer *et al*, which used acute labeling with intravenous fluorescent antibodies to detect intravascular CD8^+^ T cells during ECM [20]. We have utilized a genetically encoded fluorescent protein, expressed in all T cells, coupled with the phenotypic analysis of T cells in the CNS to reveal a large population of extravasated, but still perivascular, CD8^+^ T cells that were previously unappreciated.

Interestingly, we observed accumulation of CD8^+^ T cells in the perivascular spaces of the brain during both ECM-inducing and non-inducing infections, indicating that the presence of perivascular T cells per se is insufficient to cause ECM. Instead, dynamic time-lapse movies revealed distinct behaviors of these cells in the perivascular compartment during the different infections, suggesting that it is T cell motility in the brain that correlates with their pathogenic activity during malaria infection. Infection with ECM-causing parasites resulted in a higher proportion of perivascular T cells, including Ag-specific CD8^+^ T cells that directly contribute to ECM, exhibiting behavior consistent with immunological synapse formation, including lower mean speed, higher mean arrest coefficient and lower mean confinement ratio. Whilst, further work is required to definitively resolve whether T cells interact with other cells within the brain during ECM through classical or atypical synaptic associations, these findings demonstrate the utility of two-photon microscopy in revealing information on important dynamic cellular behaviors of leukocytes that contribute to disease.

Perivascular CD8^+^ T cells were observed forming stable interactions with CX_3_CR1^+/GFP^ cells, specifically during infection with ECM-causing *Pb* ANKA parasites. Such interactions between perivascular APCs and T cells, leading to secondary *in situ* reactivation, are known to be essential for the development of pathology in other neuro-inflammatory models such as EAE [43, 44]. However, perturbation studies using clodronate liposomes and the CX_3_CR1/iDTR system [51] to deplete CX_3_CR1^+^ cells demonstrated that this interaction is not critical for development of ECM. Thus, CD8^+^ T cells mediate ECM via interaction with other non-myeloid cell populations within the brain. These results extend previously published data that peripheral myeloid cells are not required for terminal ECM development [17, 19, 48, 49] to also show that resident or recruited myeloid cells positioned behind the tight endothelial barrier of the cerebral vasculature do not non-redundantly contribute to ECM pathogenesis. This difference between EAE and ECM is perhaps unsurprising when the location of disease-associated tissue damage is considered. Whilst in EAE, perivascular T cells must receive further stimulation to cross the glia limitans into the parenchyma, where the disease-associated pathology occurs [27, 56], in malaria the ECM-associated pathology is focused primarily to the cerebral vasculature [13], which is accessible to both luminal and perivascular CD8^+^ T cells. However, in contrast to luminal cells, it is also possible that arrested perivascular CD8^+^ T cells may exert a secondary activity during ECM by interacting with the glia limitans. This may induce signals into the parenchyma of the brain, contributing to the activation and injury of brain-resident cells including astrocytes, microglia and neurons, which is observed during ECM [57–59].

Thus, the critical questions are 1) why do T cells behave differently in the brains of ECM-inducing and non-inducing malaria infections, and 2) why can they specifically cause cerebral pathology only during *Pb* ANKA infection? We found that intracerebral CD8^+^ T cells possess an equally activated phenotype in mice with ECM-causing and non-ECM causing infections, indicating that upon migration to the brain, CD8^+^ T cells possess the intrinsic ability to mediate cerebral pathology, as long as they receive the necessary tissue signal. This result is in agreement with Howland *et al*. who reported that parasite specific CD8^+^ T cells with cytolytic potential [26] are found within the brains of mice infected with non-ECM causing parasites. Indeed, it is likely that T cells recruited to the brain during *Pb* ANKA and NK65 infections exhibit comparable antigen specificities, as a number of the most immunogenic antigens are conserved between *P*b ANKA and other murine *Plasmodium* parasites [60, 61]. Moreover, CD8^+^ T cells specific for EIYIFTNI (F4: replication protein A1), IITDFENL (Pb2: bergheilysin) and SQLLNAKYL (Pb1: PbGAP50) epitopes are observed at similar frequencies in the brains of *Pb* NK65 and *Pb* ANKA infected mice [26, 61]. Thus, Howland *et al*. suggested that the ability of CD8^+^ T cells to mediate pathology specifically during *Pb* ANKA infection is the presentation of parasite antigen by microvessel endothelial cells in the brain during this infection. This hypothesis is consistent with Haque *et al*.*’s* report that antigen-specific CD8^+^ T cells migrate to the brain, but do not induce ECM until a critical antigen threshold is reached within the brain [11].

The source of malarial antigen and its mode of presentation to pathogenic T cells specifically during ECM-inducing malaria infections has been a subject of intense investigation. A number of studies have shown that, despite similar peripheral parasitemia in the blood, parasite accumulation within the brain is higher during infection with ECM-causing parasites than non-ECM causing parasites [26, 37]. Similarly, greater parasite accumulation is found in the brains of ECM-susceptible strains of mice than resistant strains [37]. In agreement with these studies, we observed significantly higher accumulation of *Pb* ANKA parasites than *Pb* NK65 parasites within the brain on day 7 of infection, when *Pb* ANKA infected mice developed ECM. Whilst these data support a major role for parasite accumulation in the brain in ECM-pathogenesis, *Pb* ANKA parasite accumulation was infrequent compared with the level of *P*. *falciparum* sequestration observed during CM. Moreover, consistent with Nacer et al [13], we failed to observe high levels of adhesion of luminal parasites to brain vascular endothelium during *Pb* ANKA infections by intravital imaging. Thus, although *Pb* ANKA sequestration does not lead to vessel occlusion during ECM, accumulation of pRBCs within the brain, and specifically within the perivascular space, may provide a localized source of antigen for cross-presentation by endothelial or associated cells during *Pb* ANKA infection, enabling perivascular CD8^+^ T cells to mediate their pathogenic activity. Notably very recently, Howland *et al*. [53] have also shown that brain endothelial cells are significantly more efficient at cross presenting *Pb* ANKA parasites than *Pb* NK65 or *P*. *yoelii* NL parasites, providing a further mechanism for increased antigen expression with the brain during *Pb* ANKA infection than in other non-ECM inducing infections. Detailing the properties and features of *Pb* ANKA parasites that cause them to reproducibly promote ECM, unlike other *Pb* isolates, require further investigation, especially as there appears to be little genetic polymorphism between them [15, 16].

Irrespective of how CD8^+^ T cells encounter antigen within the brain during *Pb* ANKA infection, the question remains as to the mechanism through which they mediate ECM pathology. The requirement for perforin and granzyme B in CD8^+^ T cell-mediated disruption of cerebral vascular integrity, leading to ECM, has previously led to the hypothesis that CD8^+^ T cells directly induce apoptosis of endothelial cells within the brain [11, 24, 25]. In support of this, perivascularly located T cells were closely associated with the abluminal surface of blood vessels in both infections but more so in mice infected with *Pb* ANKA than *Pb* NK65 parasites. However, although increased above levels seen in brains from uninfected mice, the number of apoptotic endothelial cells within the brains of mice with ECM appeared insufficient to account for the extensive vascular leakage, which occurs during ECM. Importantly, the low level of endothelial cell apoptosis during ECM, detected by two independent methods, was not due to rapid clearance of dying cells, as there was no widespread loss of vascular endothelial cells in brains of mice with ECM. The surprisingly low level of cellular apoptosis in the brain during ECM is potentially due to the rapid progression of the ECM syndrome and the late recruitment of relatively low numbers of activated CD8^+^ T cells. Thus, as CTLs appear to locate target cells through a stochastic search strategy, and CTL immunological synapses last between 30 minutes and 6 hours [62, 63], each individual recruited CTL may target and kill a limited number of endothelial cells before the animal succumbs to infection. Consequently, our results support a model whereby vascular leakage during ECM predominantly occurs at the level of the interendothelial tight junctions without, or long before, causing endothelial cell death [13]. Supporting this hypothesis, Suidan *et al*. have demonstrated the ability of intracerebral antigen-specific CD8^+^ T to initiate central nervous system vascular leakage through a perforin-dependent mechanism involving VEGF, that down-regulates tight junction proteins 12–24 hours before activation of the apoptotic caspase cascade [64]. Notably, VEGF is increased in the brains of mice with ECM [65] and humans with CM [66, 67] and has been shown to inhibit endothelial cell apoptosis [68–70]. This scenario provides a potential explanation for the rapid recovery from ECM and reestablishment of the vascular integrity that can occurs after administration of anti-malarial drugs, which would not be possible if vascular dysfunction was mediated through extensive loss of endothelial cells.

**Fig 10:**
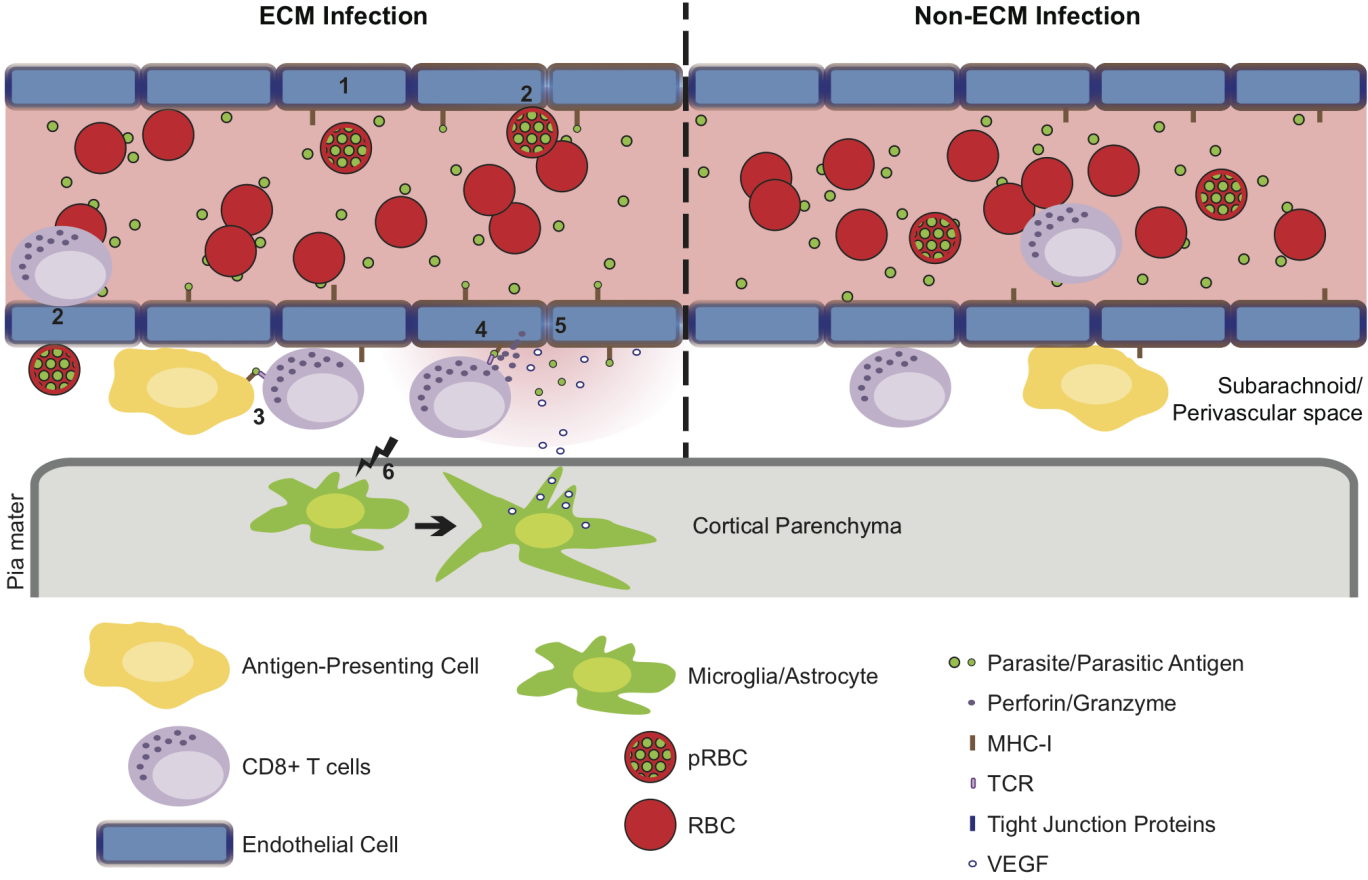
Hypothetical model for the CD8^+^ T cell-dependent development of ECM. (1) *Pb* ANKA infection leads to the upregulation of adhesion molecules and cross-presentation of parasite antigen by MHC-I on brain microvascular endothelial cells [85–87]. (2) This promotes transient interaction of *Pb* ANKA-pRBCs and rolling of activated CD8^+^ T cells on the luminal aspect of the brain microvessel endothelial cells. Beginning one day prior to signs of ECM, parasite-specific CD8^+^ T cells are recruited to the perivascular space, either via direct diapedesis across the endothelium, or migration via the highly permissive choroid plexus. In the perivascular space, parasite-specific CD8^+^ T cells form immune synapses with (3) parasite Ag-expressing APCs and (4) the basolateral membrane of cross-presenting endothelial cells. Perivascular APCs may acquire parasite antigen as a result of either transport of material across the vessel wall preceding generalized breakdown of the barrier or subsequent to breach of cerebral vascular integrity. (5) The interaction between CD8^+^ T cells and the basolateral membrane of endothelial cells leads to release of cytotoxic perforin and granzyme molecules in the perivascular space that down regulate intercellular tight junction proteins, damaging the vascular integrity and causing vasogenic edema. (6) Through an undefined mechanism, perivascular CD8^+^ T cells also communicate across the glia limitans to induce astrocyte and microglial activation. Activation of these cells further amplifies cerebral inflammation and dysfunction and provides a source of VEGF that concomitantly induces vascular leakage and resistance of endothelial cells to apoptosis. In non-ECM malarial infections, parasites do not accumulate in the brain, brain endothelial cells do not phagocytose or cross-present malaria antigen and perivascular CD8^+^ T cells fail to recognize their cognate antigen, restricting their pathogenic activity and preventing ECM development.

In summary, we have presented data that highlights the perivascular space as a site of significant CD8^+^ T cell accumulation during murine malaria infections. This site was additionally found to be a site for pRBC accumulation. Together, our results suggest that pathogenically relevant interactions between CD8^+^ T cells and their target cells, most likely cross presenting endothelial cells, may occur at the perivascular aspect of affected vessels, shifting the focus from intraluminal events to include this previously underappreciated location. We, therefore, propose that infection with malaria parasites results in accumulation of perivascular antigen-specific CD8^+^ T cells and antigen-dependent *in-situ* active engagement of the T cell receptor, only during *Pb* ANKA infection, leading to alteration of tight junction proteins, increased vascular permeability and death before the widespread occurrence of endothelial cell apoptosis **(Fig 10)**.

## Materials and Methods

### Ethics

Animal work in New York was carried out in strict accordance with the recommendation in the Guide for the Care for the Care and Use of Laboratory Animals of the Public Health Service (National Institutes of Health) and was approved by New York University School of Medicine Institutional Animal Care and Use Committee (IACUC). Animal work in the U.K. was approved following local ethical review by the Universities of Manchester and Glasgow Animal Procedures and Ethics Committees and was performed in strict accordance with the U. K Home Office Animals (Scientific Procedures) Act 1986 (approved H.O Project Licenses 70/6995 and 70/7293). All surgery was performed under anesthesia: ketamine (50 mg/kg), xylazine (10 mg/kg), acepromazine (1.7 mg/kg); or isoflurane (2% in O_2_ at 0.2 L/min).

### Mice

The following mice were used in the study: At Skirball Institute Animal facility, C57BL/6 (H-2b) from the National Cancer Institute or Taconic Labs, OT-I [71], P14 [52], CFP [72], DsRed [73], CX_3_CR1^CreER^ X Rosa26^iDTR^ mice [51]. Both CFP and DsRed were expressed under the control of a chicken β-actin promoter and CMV enhancer cassette. CX_3_CR1^CreER^ X Rosa26^iDTR^ (referred to as CX_3_CR1-iDTR) mice express Cre in CX_3_CR1^+^ cells upon treatment with tamoxifen, inducing expression of the diphtheria toxin receptor (DTR) and, thus, sensitivity to diphtheria toxin (DT) in those cells. At the Universities of Glasgow and Manchester, C57BL/6 mice from Harlan and Charles River, UK, hCD2-DsRed [74], CX_3_CR1^GFP/GFP^ [45], CX_3_CR1^GFP/GFP^ X C57BL/6 (F1) and CX_3_CR1^GFP/GFP^ X hCD2-DsRed^+/+^ mice (F1). In all cases, transgenic mice were fully backcrossed to a C57BL/6 background and were used between 6 and 12 weeks of age. Mice were maintained in specific pathogen-free conditions.

### Parasites

GFP-expressing *P*.*berghei* ANKA parasites (GFP expressed under control of elogation factor 1a [eEF1a] promoter) were a kind gift from Chris Janse (Leiden University Medical Center) [75]. GFP/SIINFEKL-expressing *P*.*berghei* ANKA (*Pb*-TG) parasites were a kind gift from William Heath (University of Melbourne) [22]. *P*.*berghei* NK65 parasites expressed GFP under control of the circumsporozoite promoter [76]. Parasites were maintained in liquid nitrogen and passaged through naive mice prior to being used to infect experimental animals. Experimental infections were initiated by i.v. inoculation with 10^4^ or 10^6^ pRBCs, depending upon the experiment, and infected mice were monitored for neurological symptoms (paralysis, ataxia, convulsions, and coma occurring between day 6 and 10 post-infection). Parasitemia was measured daily from day 3 p.i. by either examination of Giemsa-stained thin blood smears or by flow cytometric detection of DAPI stained GFP^+^ parasites **(S11 Fig)**.

### Classification of experimental cerebral malaria

The induction and severity of ECM was assessed using the following well-defined grading system [77] 1: no signs; 2: ruffled fur/and or abnormal posture; 3: lethargy; 4: reduced responsiveness to stimulation and/or ataxia and/or respiratory distress/hyperventilation; 5: prostration and/or paralysis and/or convulsions. Stages 2/3 were classified as prodromal signs of ECM and stages 4/5 were classified as ECM.

### Adoptive transfer

Splenic DsRed^+/+^OT-I CD8^+^ T cells were purified (>95% purity) from a naïve DsRed^+/+^OT-I TCR Tg mouse using a Dynal Mouse CD8^+^ Negative Isolation Kit (Invitrogen). 10^4^ and 10^6^ DsRed^+/+^OT-I CD8^+^ T cells were transferred i.v. into C57BL/6 and P14 TCR Tg recipients, respectively, one day prior to infection with 10^6^ *Pb*-TG-pRBCs, as described above.

### Detection of vascular leakage

Vascular leakage was detected as described previously, with some modifications [78]. Briefly, 50 μL 3% Evans blue/PBS (w/v) was injected i.v. into anesthetized mice and allowed to circulate for 5–6 hours. 100–150 μL blood was collected just before perfusion for serum sample. Mice were exsanguinated under KXA anesthesia by intracardial perfusion with 20 mL ice cold PBS. Each brain was removed, weighed, deposited in 500 μL formamide and incubated in darkness at 37°C for 48 hours to extract Evans blue. Formamide was then aspirated and Evans blue absorbance at 610 nm was measured for serum and brain samples with EnVision 2104 Multilabel Reader plate reader (PerkinElmer). Leakage was calculated as Evans blue concentration(brain) multiplied by extraction volume (500 μL), divided by Evans blue concentration(serum), divided by brain mass, divided by hours of circulation, yielding μL serum/g brain/hr.

### Intravital 2-photon microscopy

Non-recoverable intravital transcranial imaging was performed utilizing adapted published protocols [79–81]. Infected mice were imaged on days 5, 6 or 7 post infection. For the imaging of mice with ECM, mice were selected only when they scored 3 or above using the grading system described above. Mice imaged on day 5 post infection showed no signs of ECM. The following numbers of mice were imaged to assess the polyclonal T cell response within the subarachnoid and perivascular spaces:—2 mice infected with *Pb* ANKA on day 5 p.i., before ECM development, 4 mice with ECM (score > 3) on day 7 p.i., 4 mice infected with *Pb* NK65 on day 7 p.i. and 4 uninfected mice. To visualize blood vessels, mice were injected (i.v.) with either 20 ng Evans blue in PBS or 10 μL Qtracker 705 non-targeted quantum dots (Invitrogen) in PBS prior to imaging. After exposure of the skull by removal of the scalp and periosteum, mice were immobilized in a stereotaxic apparatus. An imaging window (∼3-mm diameter) was created on the right parietal bone 2–3 mm lateral and posterior to bregma by thinning the bone with a micro-drill, under a dissecting microscope.

Two-photon transcranial microscopy was performed with either a LSM-7 MP or LSM-710 system (Zeiss), using a 20x W Plan-Apochromat water immersion objective (NA 1.0, Zeiss). Excitation wavelengths between 910 and 940 nm were generated by a tunable Ti-sapphire femtosecond pulsed laser. Fluorescent emission signals were detected using a combination of non-descanned fluorescence detectors. Emission signals were sequentially separated by dichroic mirrors and bandpass filters arranged in two configurations: 1) 740 and 625 dichroic mirrors (Qtracker 705), 490-nm dichroic mirror with a 485-nm shortpass (SHG), and 593-nm dichroic mirror in combination with 525/25 (GFP) and 585/22 (DsRed) bandpass filters (Semrock). 2) 442/45 (SHG) filter, 465-nm dichroic mirror with 483/35 (CFP/GFP) filter, 505-nm dichroic mirror with 538/36 (GFP/CellEvent) filter, 555-nm dichroic mirror with 610/70 (DsRed) filter and 660-nm dichroic mirror with 710/100 (Evans blue-albumin) filter (Chroma or Semrock). CFP and GFP signals were distinguished by assessing the ratio of 483/35 signal to 538/36 signal. Mice were maintained under anaesthesia throughout surgical and transcranial imaging procedures. Core body temperature was maintained at 37°C, thermostatically controlled by a rectal temperature probe. Perfusion of the cranial window with an isotonic solution was maintained throughout the imaging and provided a meniscus for the dipping lens objective. Preparation and imaging of an individual animal lasted up to 3 hours. Individual movies lasted between 15 and 35 minutes for uninfected and Plasmodium infected mice. For LCMV infected mice, movies lasted up to 1 hour. Imaging sessions started with the generation of a tile scan (1416x1416 μm) of the area in the center of the imaging window. This ‘map’ of the visible area included an average of 8.8±2.1 large vessels (30–100 μm) along with their associated smaller ancillary vessels. Further image acquisition was centered on these larger vessels, in non-overlapping regions of the tile scan. Vessels with T cells visible in the initial tile scan were selected for further image acquisition for motility analyses.

### Two-photon image processing and analysis

Two-photon time-lapse sequences acquisition (283x283x30 μm, 2 μm Z step) (Zen software, Zeiss) was performed with low laser power and short pixel dwell time. As a result, significant dark current shot noise was present in some of our images, which had a detrimental impact on our chosen automated tracking software. In order to eliminate this high frequency speckled noise, a multiscale, -undecimated "A Trous" wavelet transform [82] based on a 3x3x3 linear kernel was applied on each volume of interest. Each channel and each time-point were decomposed separately into 7 additive layers plus final residual layer. Noise was mostly contained in the first layer, and cells were best detected in the 4th layer **(S12 Fig)**. By selecting the layers containing only cells, we produced time-lapse sequences which were devoid of noise and therefore suitable for cell tracking. Similarly, the blood vessel stacks were filtered by summing layers 3 to 7 of the wavelet decomposition. Values below 0 were ignored and a volumetric distance map was obtained by successive greyscale dilations and summations of this new image stack using a 3x3x3 structuring element. In the distance map, pixel values are proportional to the distance from the blood vessels and 0 inside the blood vessels. The volumetric distance map was used both to discard any cells inside the blood vessels, and for the remaining cells, to measure their distance from the blood vessels and perform motility analyses **(S13 Fig)**. Imaris (Bitplane) or Volocity (Improvision) software packages were used track cells using a combination of automated and manual processing. Motility analyses were subsequently exported and processed in Excel. Mean speed was calculated as path length/time (μm/min). Mean instantaneous speed was calculated by determining speed of a tracked cell at each consecutive time-point. Arrest coefficient of a cell was defined as the percentage of time points when its instantaneous speed was <2 μm/min. The confinement ratio was calculated as track displacement (distance between start and end point)/track length. Cellular contacts were determined manually in 3D to determine a lack of space between interacting cells at each time point. Analyses were performed for cells with tracks of at least 5 time-points.

### Flow cytometry

Brain sequestered leukocytes were isolated from PBS perfused mice as previously described [83]. Isolated brain leukocytes were surface stained with α-mCD8 (53–6.7), CD45 (30-F11), CD3 (17A2), CD11a (M17/4), CD11b (M1/70), CD69 (H1.2F3), CXCR3 (CXCR3-173), KLRG1 (2F1), CD44 (IM7), CD62L (MEL-14), ICOS (15F9), CD40 (3/23), CD80 (16-10A1), MHC I (28-8-6), CD19 (1D3), CD49b (DX5). Intracellular staining for granzyme B (GB11) was performed for 1 hr, after treatment with fix-perm (eBioscience). Dead cells were excluded using forward scatter and side scatter properties and/or LIVE/DEAD Fixable Blue Dead Cell Stain Kit (Life Technologies). Fluoresce minus one controls were used to set gates. Cells were analysed with a BD LSR II (Becton Dickinson) or MACSQuant (Miltenyi) using BD FACSDiva software (Becton Dickinson), and data was analyzed with FlowJo (Tree Star Inc.). All antibodies were from eBioscience and Biolegend

### Systemic and intraventricular depletion of macrophages

Macrophages were depleted during infection by the administration of clodronate-liposomes. Systemic depletion was performed from day 5 p.i. by injection (i.p.) of 300 μL clodronate liposomes. Depletion of perivascular macrophages, which are present behind the blood vessel wall and are not depleted by systemic administration of clodronate liposomes [84], was performed on day 5 p.i. by i.c.v. injection of 8 μL clodronate liposomes into the right lateral ventricle, adapted from Galea *et al*. [50]. Briefly, animals were anesthetized with isoflurane (2%) in O_2_ (0.2 L/min) and N_2_O (0.4 L/min) and craniectomy performed. I.c.v. injection was performed using a glass microneedle (co-ordinates from bregma: anterior–posterior -0.22 mm, lateral −1.0 mm, ventral −2 mm. Sub-cutaneous buprenorphine was prophylactically administered at 0.1 mg/kg. The i.c.v. protocol was optimized and the injection placement within the ventricle confirmed by assessing the circulation of trypan blue throughout the perivascular compartments.

### Systemic depletion of CX3CR1 cells

CX_3_CR1^+^ cells were depleted from *Pb* ANKA infected mice as follows: CX_3_CR1-iDTR mice were initially infected with 10^6^ *Pb* ANKA pRBCs i.v. On day 1 and 2 p.i., infected mice were treated daily with 5 mg tamoxifen dissolved in corn oil via oral gavage. On day 3, 4 and 5 p.i., mice were treated daily with 1 μg diphtheria toxin i.p. to thoroughly deplete DTR-expressing CX_3_CR1^+^ cells. CX_3_CR1^+^ cell depletion was confirmed by brain histology of CX_3_CR1-expressing microglia **(Fig 7C)**. Mice were monitored daily for ECM symptoms and survival, as described.

### Histology

For detection of GFP^+^ parasites, CD31 (MEC 13.3, BD Pharmingen), activated caspase 3 (Asp175, Cell Signalling), CD8 (YTS105.18, AbD Serotec) and Lectin (biotinylated *Lycopersicon esculentum*, Sigma) in 30 μm thick free floating brain sections: mice were sacrificed by exposure to a rising concentration of CO_2_. Spleens were removed before sequential transcardial perfusion with PBS and 4% PFA. Brains were removed and sequentially incubated at 4°C overnight in 20% sucrose/4% PFA and 20% sucrose/PBS. Brains were snap frozen with dry ice and sectioned on a freezing sledge microtome (Bright Instruments, Cambridge, UK) at a thickness of 30μm. Brain sections were stored in cyroprotectant solution (30% ethylene glycol, 20% glycerol in PBS) at -20° C until used. Free-floating sections were washed in PBS and then blocked in 10% goat serum (Sigma Aldrich, UK) in primary diluent (PBS and 0.3% Triton X-100) for 1.5 hrs. Combinations of primary antibodies in primary diluent were applied for 12–48 hrs at 4°C. Combinations of secondary antibodies in primary diluent (goat anti-rabbit 488 and 546, Life Technologies, goat anti-rat 647, Life Technologies and Streptavidin conjugated 546, Life Technologies) were applied for 1.5 hrs at RT. Cell nuclei were counterstained with DAPI (Life Technologies, UK). Sections were mounted onto gelatine coated slides and coverslipped in Prolong Diamond anti-fade mountant (Life Technologies, UK). Images were collected on an Olympus BX51 upright microscope using 20x/ 0.50 and 40x/ 0.75 Plan Fln objective and captured using a Coolsnap ES camera (Photometrics) through MetaVue Software (Molecular Devices). Images were processed using ImageJ (http://rsb.info.nih.gov/ij). Quantitation of parasite load, vasculature and apoptosis was performed in a semi-automated fashion using Image-Pro Premier (MediaCybernetics). ImagePro’s smart segmentation technology was utilized to ascribe areas of interest (i.e parasite/apoptotic cell/vessel) and background from an image. The software then automatically determined all regions of interest from the total image; providing data regarding number of regions of interest and total area these regions occupied in the image. These settings were globally applied to all samples ensuring a non-biased analysis of all samples.

For detection of CD31 and Iba1 in fresh frozen sections: mice were exsanguinated under KXA anesthesia by intracardial perfusion with 20 mL ice cold PBS supplemented with 10 U/mL heparin. For immunohistofluorescent staining, brains were embedded in Optimum Cutting Temperature compound (O.C.T., Tissue-Tek) and frozen in a dry ice/isopropanol bath. 6–8 μm thick coronal sections were cut with a Leica CM3050 S cryotome sectioning system and mounted onto SuperFrost slides. Mounted sections were fixed in acetone at -20°C for 5 min, allowed to air dry for at least 5 hours, followed by rehydration, in TBS (20 m*M* Tris, 150 m*M* NaCl, pH 7.5) with orbital shaking for 10 min at RT. Sections were permeablized by soaking in TBST (0.05% Tween-20/TBS) with orbital shaking for 15 min at RT, then blocked by addition of 5% BSA, 10% mouse serum, TBS for 30 min at RT. Following washing in TBST sections were stained in the dark for 1 hour at RT with rat α-CD31-AlexaFluor647 (MEC13.3, Invitrogen) or rabbit α-Iba1 (Wako) antibodies diluted in TBST, and washed with TBST. The Iba1-stained tissue was further probed with goat α-rabbit-AlexaFluor647. After washing probes away, cryosections were mounted with glycerol under coverglass, and sealed with nail polish. For unstained histology, each brain was removed and fixed in 4% PFA/PBS (w/v) at 4°C overnight. Brains were washed with PBS 3 times for 5 min each. 500-μm thick coronal sections were cut with a Pelco100 vibratome sectioning system (Ted Pella Inc.) and mounted onto Superfrost slides with glycerol and sealed with nail polish. Samples were imaged with a 25x LD LCI Plan-Apochromat objective (NA 0.8, Zeiss) and analysed using Photoshop CS5 (Adobe).

### *Ex vivo* apoptosis detection

10 μL 2 m*M* CellEvent Caspase-3/7 Green Detection Reagent (Invitrogen) was injected i.v. into anesthetized mice and allowed to circulate for 30–60 minutes. Z stacks were captured by two-photon microscopy spanning a surface area of 4–5 mm^2^ and a depth of ∼150 μm. CellEvent-positive cells were counted and their number divided by the volume of brain tissue imaged.

### Statistical analysis

All statistical analyses were performed using GraphPad PRISM (GraphPad Software, USA). Comparison between two groups was made using unpaired t tests, with Welch’s correction where needed. Comparison between multiple groups was made using a one-way ANOVA with Tukey’s test for multiple comparisons. Differences in survival were analysed using the Mantel-Cox log-rank test.

## Supporting Information

S1 Fig Infection with *Pb* NK65 does not cause ECM development. C57BL/6 mice were intravenously infected with 10^4^ *Pb* ANKA or *Pb* NK65 pRBCs. Peripheral parasitaemia ± SD **(A)** and development of ECM **(B)** were monitored daily. **(C)** Representative examples of Evans blue leakage in the brains from mice infected with *Pb* ANKA and *Pb* NK65 (day 7 p.i).
(TIFF)

S2 Fig Infection with LCMV induces petechial hemorrhages. C57BL/6 mice were intracranially infected with 10^4^ PFU LCMV-Armstrong and transcranial two-photon microscopy of the meninges was performed on day 6 p.i. LCMV encephalitis induces sporadic petechial hemorrhages in the meninges, visualized with Evans blue-stained blood (red). As indicated in these time-lapsed orthogonal images, such hemorrhages (white arrow) are usually repaired quickly. Such hemorrhages were not observed in any movies of symptomatic ECM mice, which could otherwise explain the perivascular deposition of pRBCs.
(TIFF)

S3 Fig Few DsRed^+^ T cells are found within the brains of mice on day 5 p.i. with *Pb* ANKA. hCD2-DsRed C57BL/6 mice were infected with 10^4^ *Pb* ANKA. Transcranial two-photon microscopy of the meninges was performed on days 5 p.i. Maximum intensity projections from intravital two-photon microscopy movies showing few DsRed^**+**^ T cells (red) within the brains of infected mice on day 5 p.i. infection with *Pb* ANKA. Blood vessels (cyan) were visualized by i.v. injection of Qtracker non-targeted quantum dots prior to imaging. Scale bar: 30 μm.
(TIFF)

S4 Fig Few leukocytes isolated from the brains of mice infected with *Pb* ANKA or *Pb* NK65 on day 7 p.i. are NK cells or B cells. C57BL/6 mice were infected with 10^4^ *Pb* ANKA or *Pb* NK65 pRBCs. **(A)** Representative flow cytometric plots showing frequencies of CD49b^+^ NK cells and CD3^+^ T cells (gated on live leukocytes) within the brains of infected mice (day 7 p.i.). **(B)** Representative flow cytometric plots showing frequencies of CD19^+^ B cells and CD3^+^ T cells (gated on live leukocytes) within the brains of infected mice (day 7 p.i.).
(TIFF)

S5 Fig T cells from isolated meningeal vessels of *Pb* ANKA infected mice on day 7 p.i. are mainly CD8^+^. C57BL/6 mice were infected with 10^4^ *Pb* ANKA or left uninfected. Meningeal vessels were removed from the whole brains of uninfected and infected (day 7 p.i.) mice and processed for flow cytometry. Representative flow cytometric plots showing frequencies of CD4^+^ and CD8^+^ T cells (gated on live leukocytes).
(TIFF)

S6 Fig CD45^hi^CD11b^hi^ monocytes and macrophages are enriched within the meninges compared with the bulk brain. CX_3_CR1^+/GFP^ mice were infected with 10^4^ *Pb* ANKA or left uninfected. Meningeal vessels were separated from the whole brains of uninfected and infected (day 7 p.i.) mice and both parts processed for flow cytometry. Representative flow cytometric plots showing frequencies of R1—CD45^int^CD11b^hi^ microglia; R2—CD45^hi^CD11b^hi^ meningeal, perivascular macrophages and inflammatory monocytes; R3—CD45^hi^CD11b^int^ leukocytes (gated on live GFP^+^ leukocytes) within the meninges **(A)** and bulk brain **(B)**.
(TIFF)

S7 Fig Parasite specific OT-I CD8^+^ T cells are highly arrested in the brains of infected wild type and P14 hosts. 10^6^ naïve DsRed-expressing OT-I CD8^+^ T cells were adoptively transferred into C57BL/6 and P14 mice, which were infected with 10^6^ SIINFEKL-expressing *Pb*-TG pRBCs. Quantification of **(A)** average perivascular T cell speeds, **(B)** arrest coefficient (proportion of time points when instantaneous velocity is <2 μm/min) and **(C)** confinement ratio (track displacement/track length) from individual three-dimensional T cell tracks, collected from mice with ECM on day 6 p.i. (n = 3).
(TIFF)

S8 Fig Apoptotic cells are commonly observed in the brain following cerebral artery occlusion induced stroke. Representative image of apoptotic cells (green) within the cortex of a mouse subjected to middle cerebral artery occlusion induced stroke 24 hours previously. Cell nuclei are shown in blue. Scale bar 50 μm.
(TIFF)

S9 Fig Wide scale loss of cerebral vasculature does not occur during ECM. C57BL/6 mice were infected with 10^4^ *Pb* ANKA pRBCs or left uninfected. Brains were removed and processed for histological examination when *Pb* ANKA mice developed signs of ECM (day 7 p.i.). Representative images demonstrating detection of CD31 expression (red) by immunofluorescence. Cell nuclei are shown in blue. Scale bar 100 μm.
(TIFF)

S10 Fig CD8^+^ T cells accumulate in perivascular compartments in deeper brain regions during ECM. C57BL/6 mice were infected with 10^4^ *Pb* ANKA pRBCs. Brains were removed and processed for histological examination when mice developed signs of ECM. A representative image from the cortex demonstrating detection of CD8^+^ T-Cells (green) proximal but abluminal to vessels (red). Cell nuclei are shown in blue. Scale bar 100 μm.
(TIF)

S11 Fig Flow cytometric measurement of parasitemia. Blood was taken from the tails of infected mice and stained with DAPI. Parasitemia was assessed via flow cytometry. Representative dot plot showing GFP expression in all DAPI^+^ pRBCs, even prior to significant parasite replication: R1 = uninfected RBCs; R2 = DAPI^low^GFP^+^ population; which differentiate into R3 = immature and mature shizonts
(TIFF)

S12 Fig Elimination of high frequency speckled noise using a multiscale, -undecimated "A Trous" wavelet transform. "A trous" wavelet decomposition of a 512x512x16 volume (time point 0) using a 3x3x3 linear kernel. A single Z slice is shown through 7 decomposition layers and residual low pass layer.
(TIFF)

S13 Fig Masking of intravascular cells for specific tracking of perivascular cells. Blood vessels (channel 1) were filtered by summing "A trous" layers 3 to 7 and thresholded to create a binary mask. DsRed T cells (channel 2) and GFP CX_3_CR_1_ cells (channel 3) were band-pass filtered using "A trous" layer 4 and pixel values below the set threshold were clipped. Cells within the blood vessel binary mask were ignored for specific tracking of perivascular cells. In the distance map, pixel values are proportional to the distance from the blood vessels and 0 inside the blood vessels. White pixels denote areas furthest from vessels (black pixels).
(TIFF)

S1 Video Transient cytoadhesion of intravascular *Pb* ANKA-pRBCs. A representative example (at ∼15 min) of a *Pb* ANKA-pRBC (green, highlighted by white circle) briefly adhering to the DsRed^+^ endothelium (orange) of an Evans blue-labeled (red) capillary, located just underneath the pia mater/glia limitans. Scale bar: 50 μm. Movie length: 31 min.
(MOV)

S2 Video Occurrence of perivascular pRBCs. A representative example of an unmoving *Pb* ANKA-pRBC (green, highlighted by white circle) just next to the DsRed^+^ endothelium (orange) of an Evans blue-labeled blood (red) vessel. Scale bar: 20 μm. Movie length: 16 min.
(MOV)

S3 Video Infection with LCMV induces petechial hemorrhages. LCMV-specific CFP^+^ P14 T cells were adoptively transferred into C57BL/6 mice, then intracranially infected with 10^4^ PFU LCMV-Armstrong and transcranial two-photon microscopy of the meninges was performed on day 6 p.i. A representative example of a petechial hemorrhage (at ∼45 min) of an Evans blue-labeled blood (red) vessel, in the context of P14 T cell (blue) movement. Scale bar: 50 μm. Movie length: 57 min.
(MOV)

S4 Video Perivascular location and arrested behavior of T cells during *Pb* ANKA infection. Representative maximum intensity projection time-lapse sequences showing accumulation and arrested behavior of DsRed T cells (red) around the *Pb* ANKA-pRBC (green)-containing and Qtracker non-targeted quantum dot-labeled blood (cyan) vessels in the meninges of *Pb* ANKA-infected hCD2-DsRed C57BL/6 mice (day 7 p.i.). Movie length: 17 min. Z step 2 μm.
(AVI)

S5 Video Additional example of perivascular location and arrested behavior of T cells during *Pb* ANKA infection. Representative maximum intensity projection time-lapse sequences showing accumulation and arrested behavior of DsRed T cells (red) around the *Pb* ANKA-pRBC (green)-containing and Qtracker non-targeted quantum dot-labeled blood (cyan) vessels in the meninges of *Pb* ANKA-infected hCD2-DsRed C57BL/6 mice (day 7 p.i.). Movie length: 17 min. Z step 2 μm.
(AVI)

S6 Video Perivascular location and dynamic behavior of T cells during *Pb* NK65 infection. Representative maximum intensity projection time-lapse sequence showing accumulation and dynamic behavior of DsRed T cells (red) around the Qtracker non-targeted quantum dot-labeled blood (cyan) vessels in the meninges of *Pb* NK65-infected hCD2-DsRed C57BL/6 mice (day 7 p.i.). Movie length: 17 min. Z step 2 μm.
(AVI)

S7 Video Additional example of perivascular location and dynamic behavior of T cells during *Pb* NK65 infection. Representative maximum intensity projection time-lapse sequence showing accumulation and dynamic behavior of DsRed T cells (red) around the Qtracker non-targeted quantum dot-labeled blood (cyan) vessels in the meninges of *Pb* NK65-infected hCD2-DsRed C57BL/6 mice (day 7 p.i.). Movie length: 17 min. Z step 2 μm.
(AVI)

S8 Video Perivascular DsRed T cells form stable interactions with CX_3_CR1^+/GFP^ cells in the brains of mice infected with *Pb* ANKA. A representative maximum intensity projection time-lapse sequence showing stable interactions between perivascular DsRed T cells (red) and CX_3_CR1^+/GFP^ cells (green) in the meninges of *Pb* ANKA infected dual-reporter mice (day 7 p.i.). Movie length: 17 min. Z step 2 μm.
(AVI)

S9 Video Perivascular DsRed T cells make contact, but do not form stable interactions, with CX_3_CR1^+/GFP^ cells in the brains of mice infected with *Pb* NK65. A representative maximum intensity projection time-lapse sequence showing brief contact made between perivascular DsRed T cells (red) and a CX_3_CR1^+/GFP^ cells (green) in the meninges of *Pb* NK65 infected dual-reporter mice (day 7 p.i.). Movie length: 17 min. Z step 2 μm.
(AVI)

S10 Video Perivascular DsRed T cells form stable interactions with CX_3_CR1^+/GFP^ cells in the brains of mice infected with *Pb* ANKA. A representative 3D cropped time-lapse sequence showing a stable interaction between a perivascular DsRed T cell (red) and a CX_3_CR1^+/GFP^ cell (green) in the meninges of a *Pb* ANKA infected dual-reporter mouse. Movie length: 17 min. Z step 2 μm.
(AVI)

S11 Video Perivascular DsRed antigen-specific T cells are mostly arrested in the brains of mice infected with *Pb* ANKA. A representative time-lapse sequence showing stably arrested DsRed OT-I T cells (orange) surrounding a subarachnoid Evans blue-labeled blood (red) vessel, and several of which are in direct contact with endothelial cells (blue). Scale bar: 50 μm. Movie length: 33 min.
(MOV)
