# Supplementary figures and images for: Perivascular Arrest of CD8+ T Cells Is a Signature of Experimental Cerebral Malaria
Source: PLoS Pathog. 2015 Nov 12;11(11):e1005210. doi: 10.1371/journal.ppat.1005210 (PMC4643016; doi:10.1371/journal.ppat.1005210)

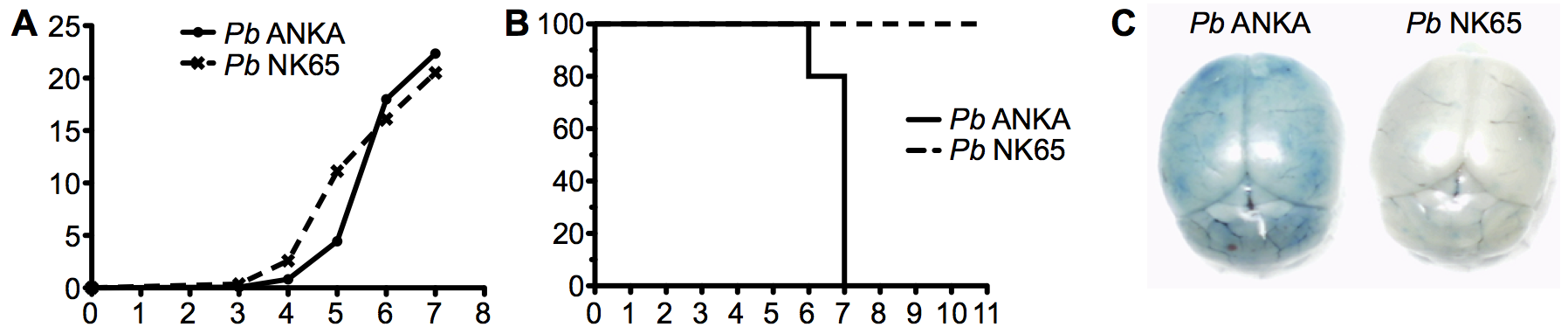

Supplement: S1 Fig — C57BL/6 mice were intravenously infected with 104 Pb ANKA or Pb NK65 pRBCs. Peripheral parasitaemia ± SD (A) and development of ECM (B) were monitored daily. (C) Representative examples of Evans blue leakage in the brains from mice infected with Pb ANKA and Pb NK65 (day 7 p.i). (TIFF) [file ppat.1005210.s001.tiff]

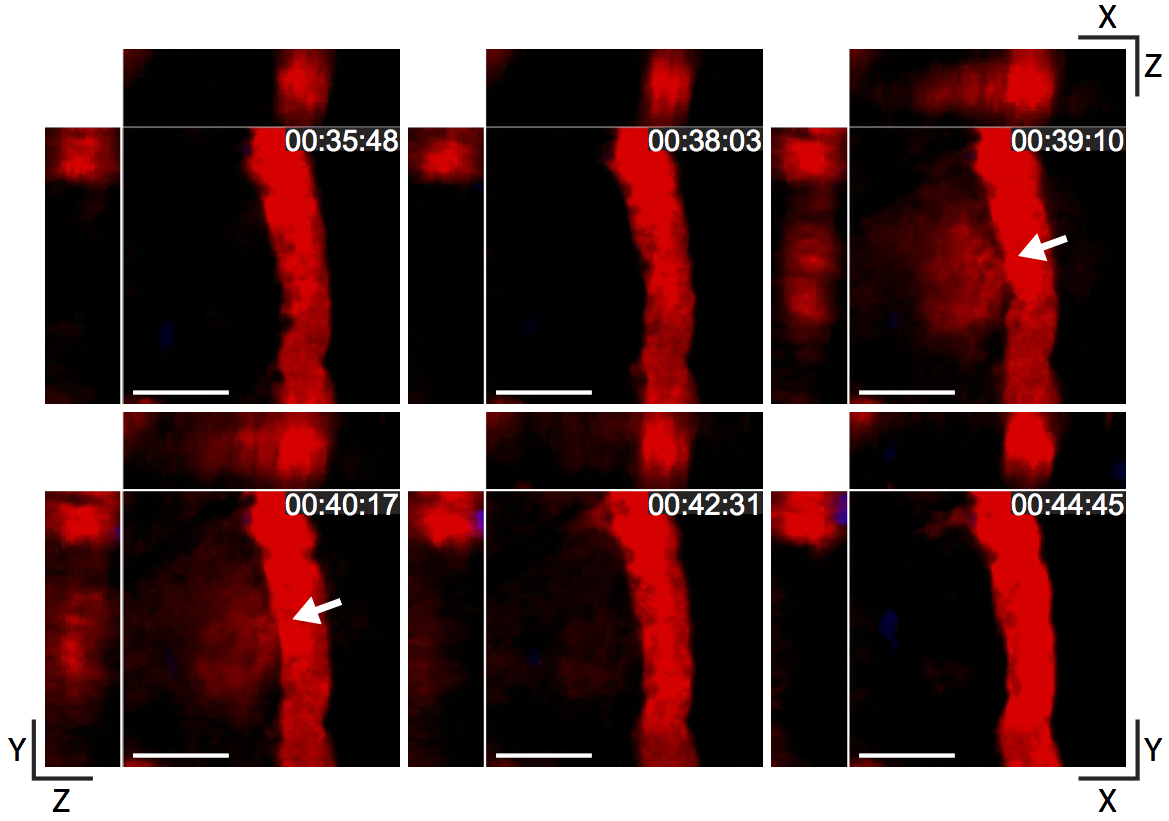

Supplement: S2 Fig — C57BL/6 mice were intracranially infected with 104 PFU LCMV-Armstrong and transcranial two-photon microscopy of the meninges was performed on day 6 p.i. LCMV encephalitis induces sporadic petechial hemorrhages in the meninges, visualized with Evans blue-stained blood (red). As indicated in these time-lapsed orthogonal images, such hemorrhages (white arrow) are usually repaired quickly. Such hemorrhages were not observed in any movies of symptomatic ECM mice, which could otherwise explain the perivascular deposition of pRBCs. (TIFF) [file ppat.1005210.s002.tiff]

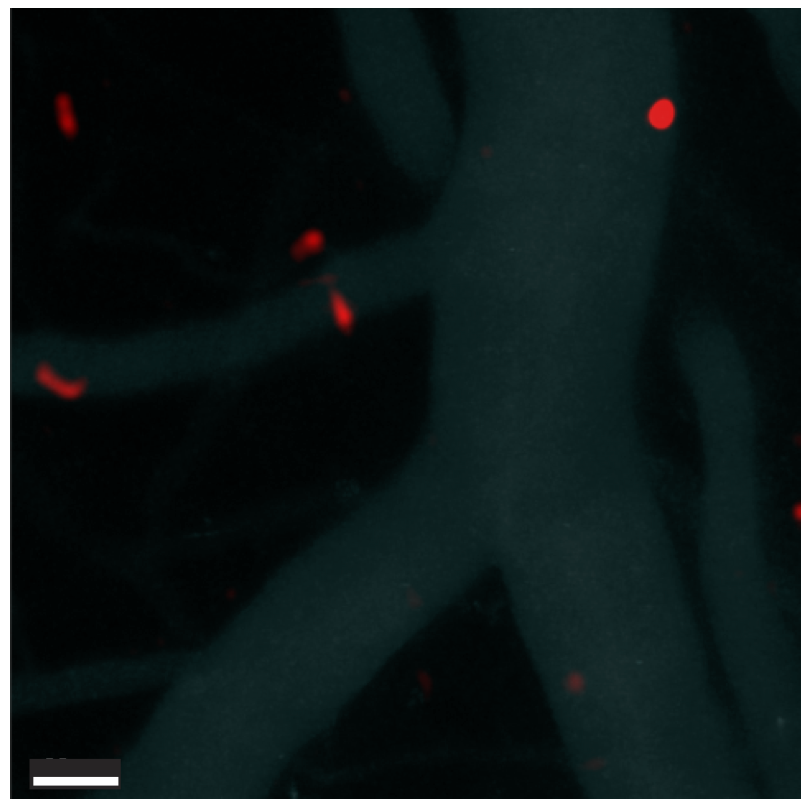

Supplement: S3 Fig — hCD2-DsRed C57BL/6 mice were infected with 104 Pb ANKA. Transcranial two-photon microscopy of the meninges was performed on days 5 p.i. Maximum intensity projections from intravital two-photon microscopy movies showing few DsRed+ T cells (red) within the brains of infected mice on day 5 p.i. infection with Pb ANKA. Blood vessels (cyan) were visualized by i.v. injection of Qtracker non-targeted quantum dots prior to imaging. Scale bar: 30 μm. (TIFF) [file ppat.1005210.s003.tiff]

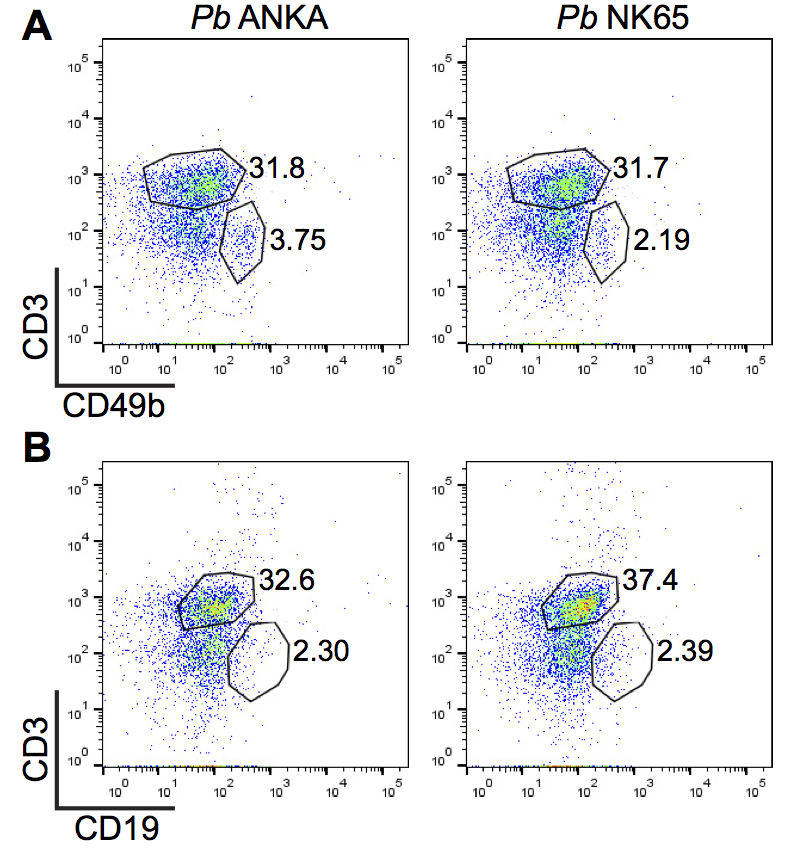

Supplement: S4 Fig — C57BL/6 mice were infected with 104 Pb ANKA or Pb NK65 pRBCs. (A) Representative flow cytometric plots showing frequencies of CD49b+ NK cells and CD3+ T cells (gated on live leukocytes) within the brains of infected mice (day 7 p.i.). (B) Representative flow cytometric plots showing frequencies of CD19+ B cells and CD3+ T cells (gated on live leukocytes) within the brains of infected mice (day 7 p.i.). (TIFF) [file ppat.1005210.s004.tiff]

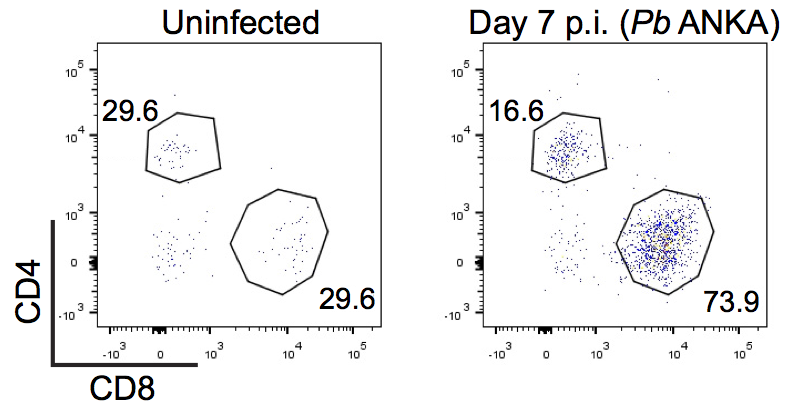

Supplement: S5 Fig — C57BL/6 mice were infected with 104 Pb ANKA or left uninfected. Meningeal vessels were removed from the whole brains of uninfected and infected (day 7 p.i.) mice and processed for flow cytometry. Representative flow cytometric plots showing frequencies of CD4+ and CD8+ T cells (gated on live leukocytes). (TIFF) [file ppat.1005210.s005.tiff]

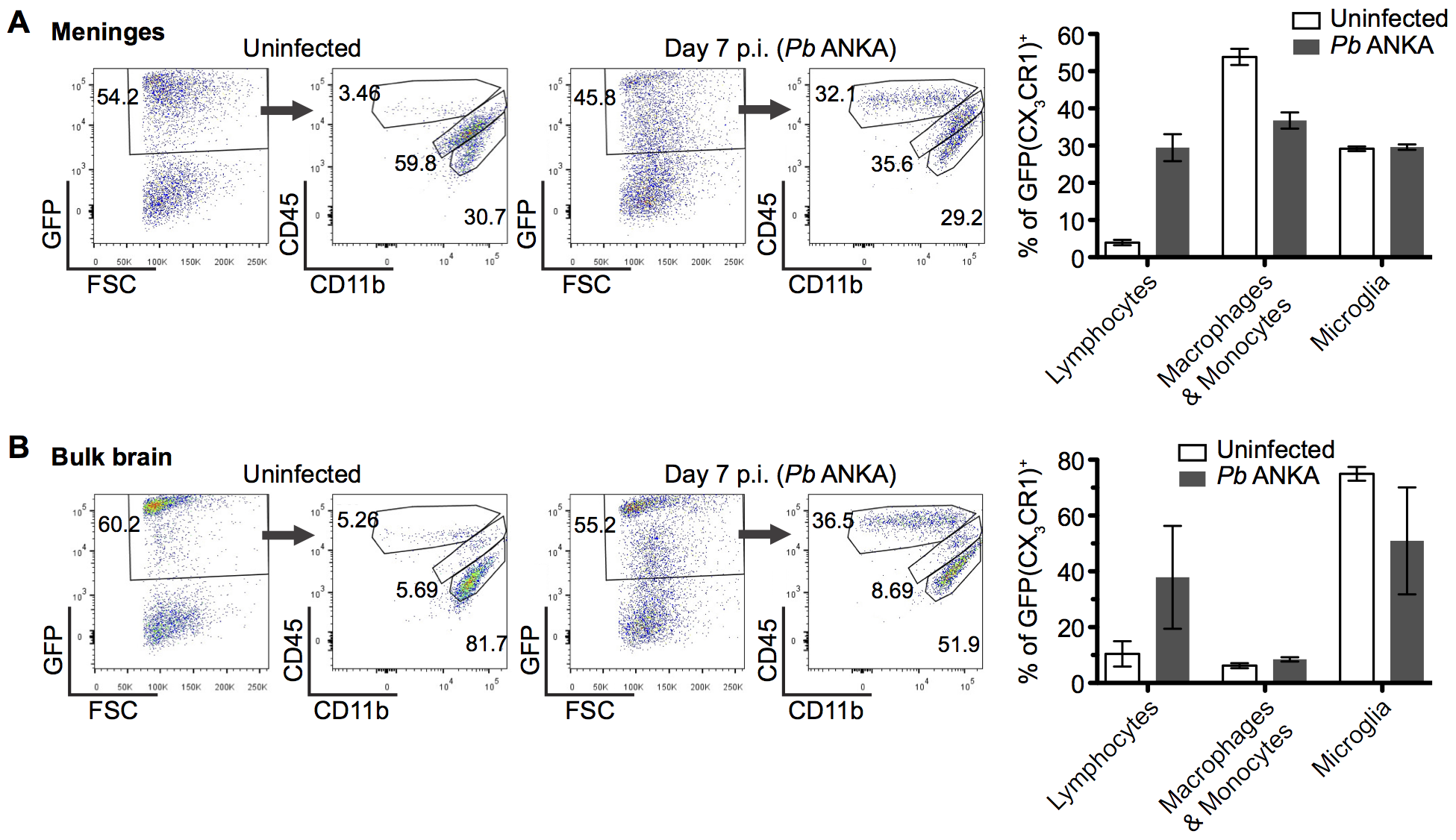

Supplement: S6 Fig — CX3CR1+/GFP mice were infected with 104 Pb ANKA or left uninfected. Meningeal vessels were separated from the whole brains of uninfected and infected (day 7 p.i.) mice and both parts processed for flow cytometry. Representative flow cytometric plots showing frequencies of R1—CD45intCD11bhi microglia; R2—CD45hiCD11bhi meningeal, perivascular macrophages and inflammatory monocytes; R3—CD45hiCD11bint leukocytes (gated on live GFP+ leukocytes) within the meninges (A) and bulk brain (B). (TIFF) [file ppat.1005210.s006.tiff]

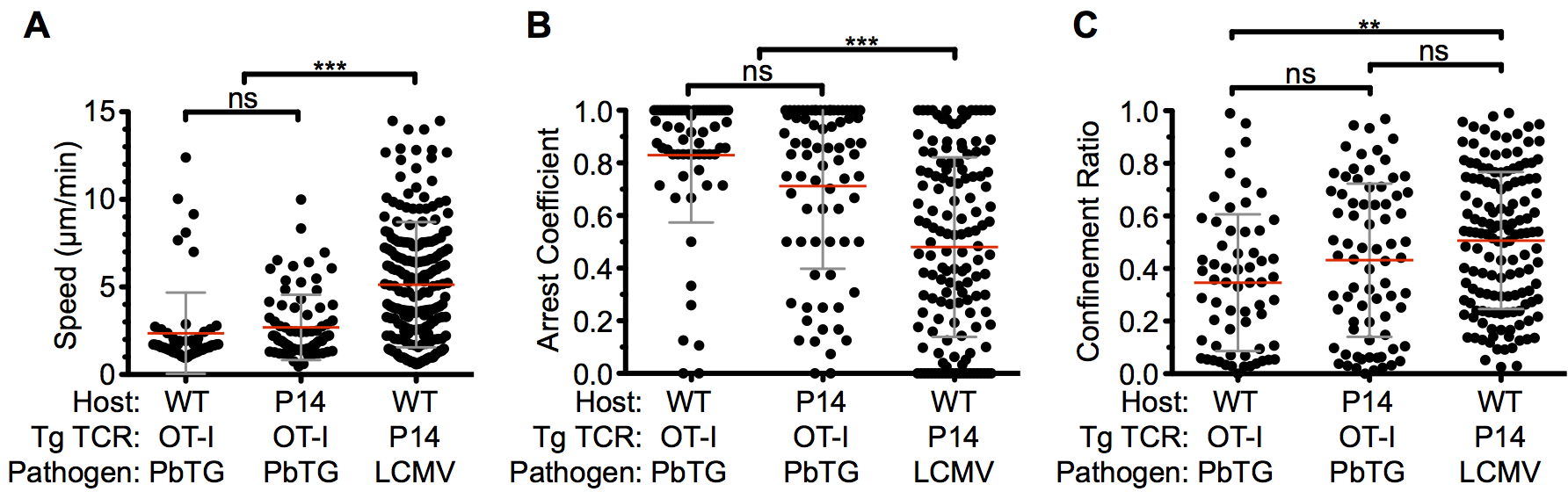

Supplement: S7 Fig — 106 naïve DsRed-expressing OT-I CD8+ T cells were adoptively transferred into C57BL/6 and P14 mice, which were infected with 106 SIINFEKL-expressing Pb-TG pRBCs. Quantification of (A) average perivascular T cell speeds, (B) arrest coefficient (proportion of time points when instantaneous velocity is <2 μm/min) and (C) confinement ratio (track displacement/track length) from individual three-dimensional T cell tracks, collected from mice with ECM on day 6 p.i. (n = 3). (TIFF) [file ppat.1005210.s007.tiff]

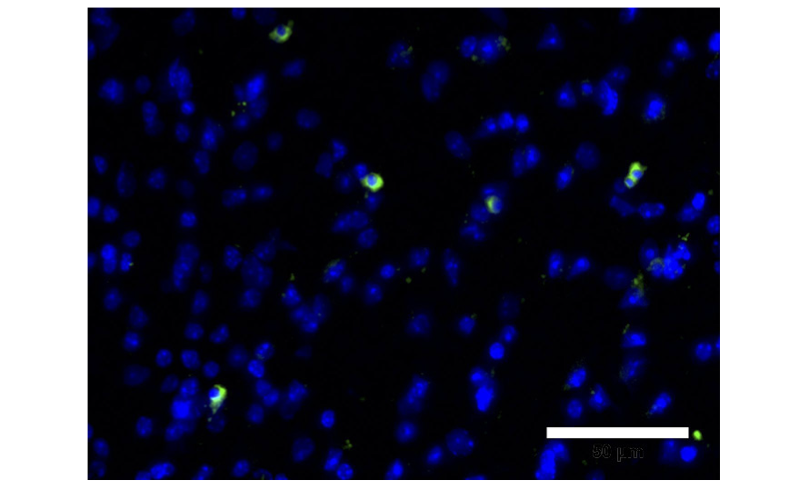

Supplement: S8 Fig — Representative image of apoptotic cells (green) within the cortex of a mouse subjected to middle cerebral artery occlusion induced stroke 24 hours previously. Cell nuclei are shown in blue. Scale bar 50 μm. (TIFF) [file ppat.1005210.s008.tiff]

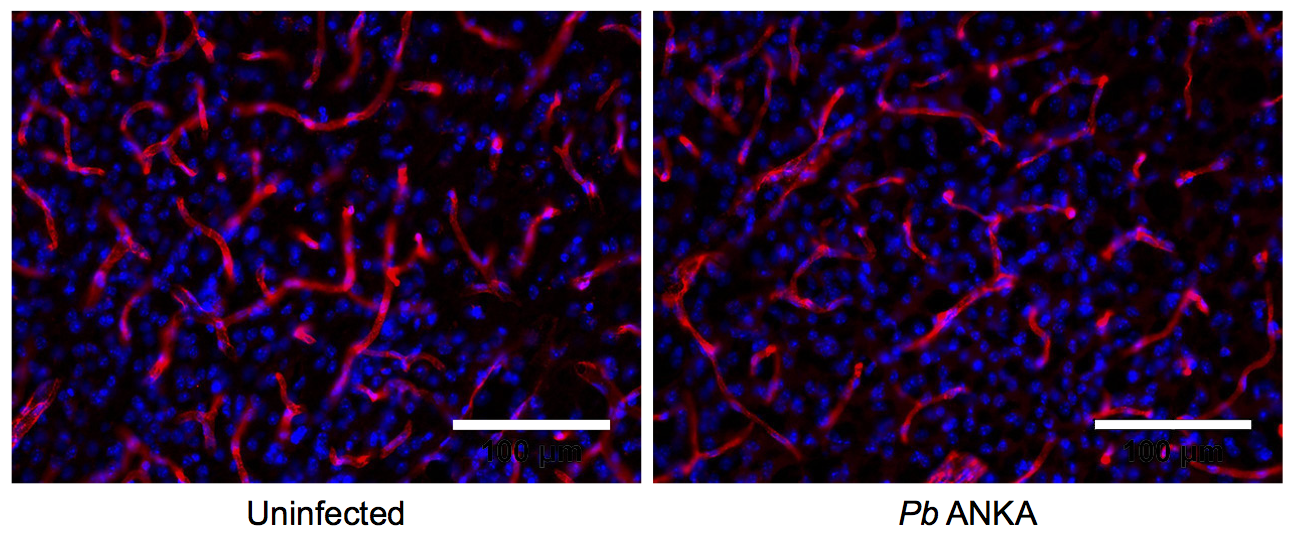

Supplement: S9 Fig — C57BL/6 mice were infected with 104 Pb ANKA pRBCs or left uninfected. Brains were removed and processed for histological examination when Pb ANKA mice developed signs of ECM (day 7 p.i.). Representative images demonstrating detection of CD31 expression (red) by immunofluorescence. Cell nuclei are shown in blue. Scale bar 100 μm. (TIFF) [file ppat.1005210.s009.tiff]

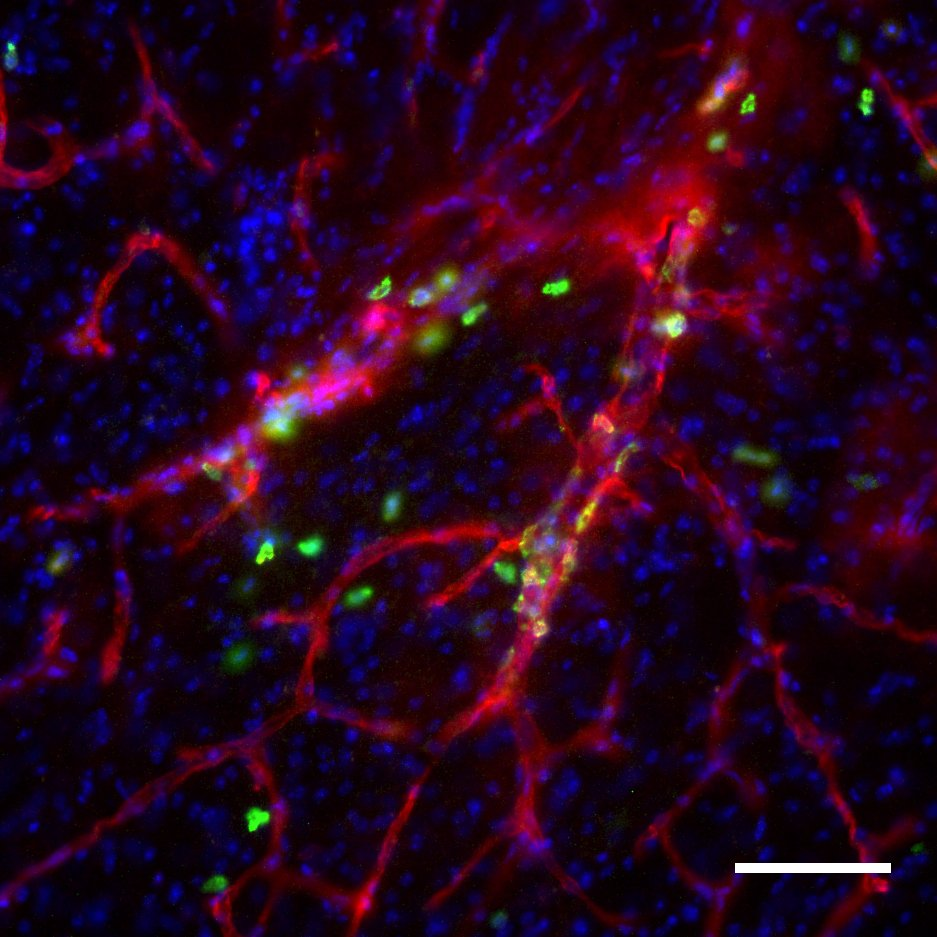

Supplement: S10 Fig — C57BL/6 mice were infected with 104 Pb ANKA pRBCs. Brains were removed and processed for histological examination when mice developed signs of ECM. A representative image from the cortex demonstrating detection of CD8+ T-Cells (green) proximal but abluminal to vessels (red). Cell nuclei are shown in blue. Scale bar 100 μm. (TIF) [file ppat.1005210.s010.tif]

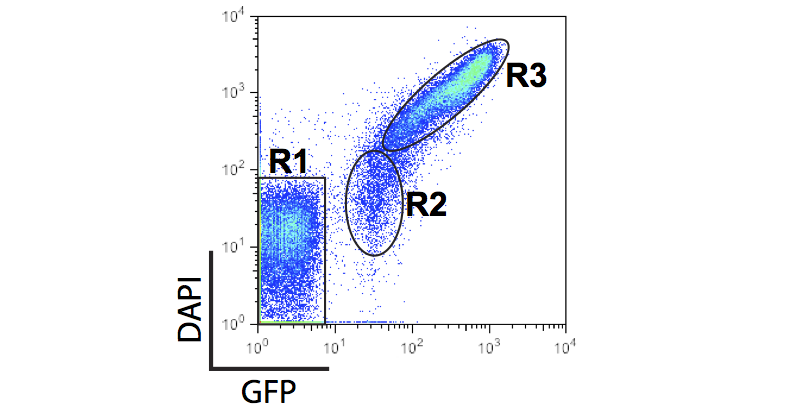

Supplement: S11 Fig — Blood was taken from the tails of infected mice and stained with DAPI. Parasitemia was assessed via flow cytometry. Representative dot plot showing GFP expression in all DAPI+ pRBCs, even prior to significant parasite replication: R1 = uninfected RBCs; R2 = DAPIlowGFP+ population; which differentiate into R3 = immature and mature shizonts (TIFF) [file ppat.1005210.s011.tiff]

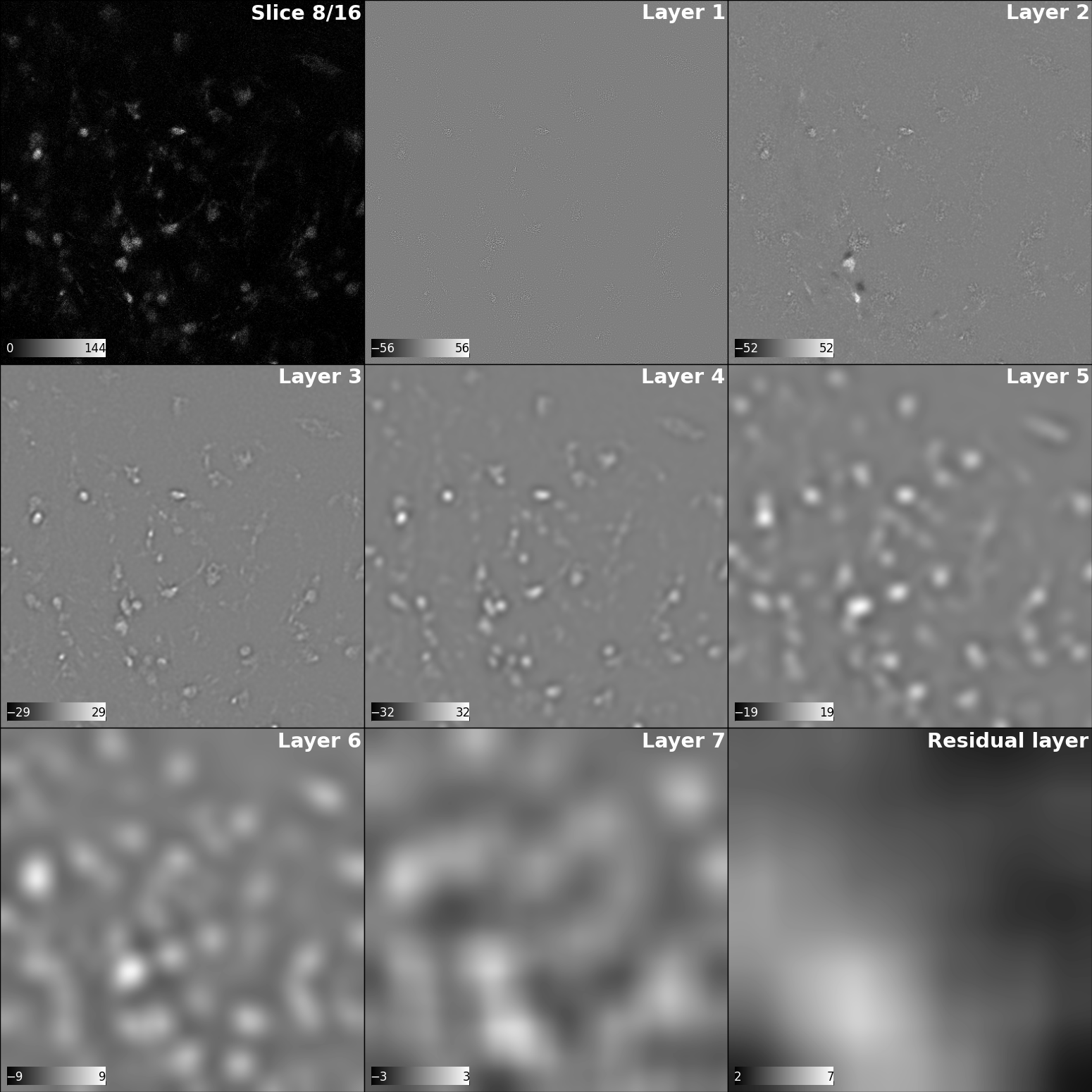

Supplement: S12 Fig — "A trous" wavelet decomposition of a 512x512x16 volume (time point 0) using a 3x3x3 linear kernel. A single Z slice is shown through 7 decomposition layers and residual low pass layer. (TIFF) [file ppat.1005210.s012.tiff]

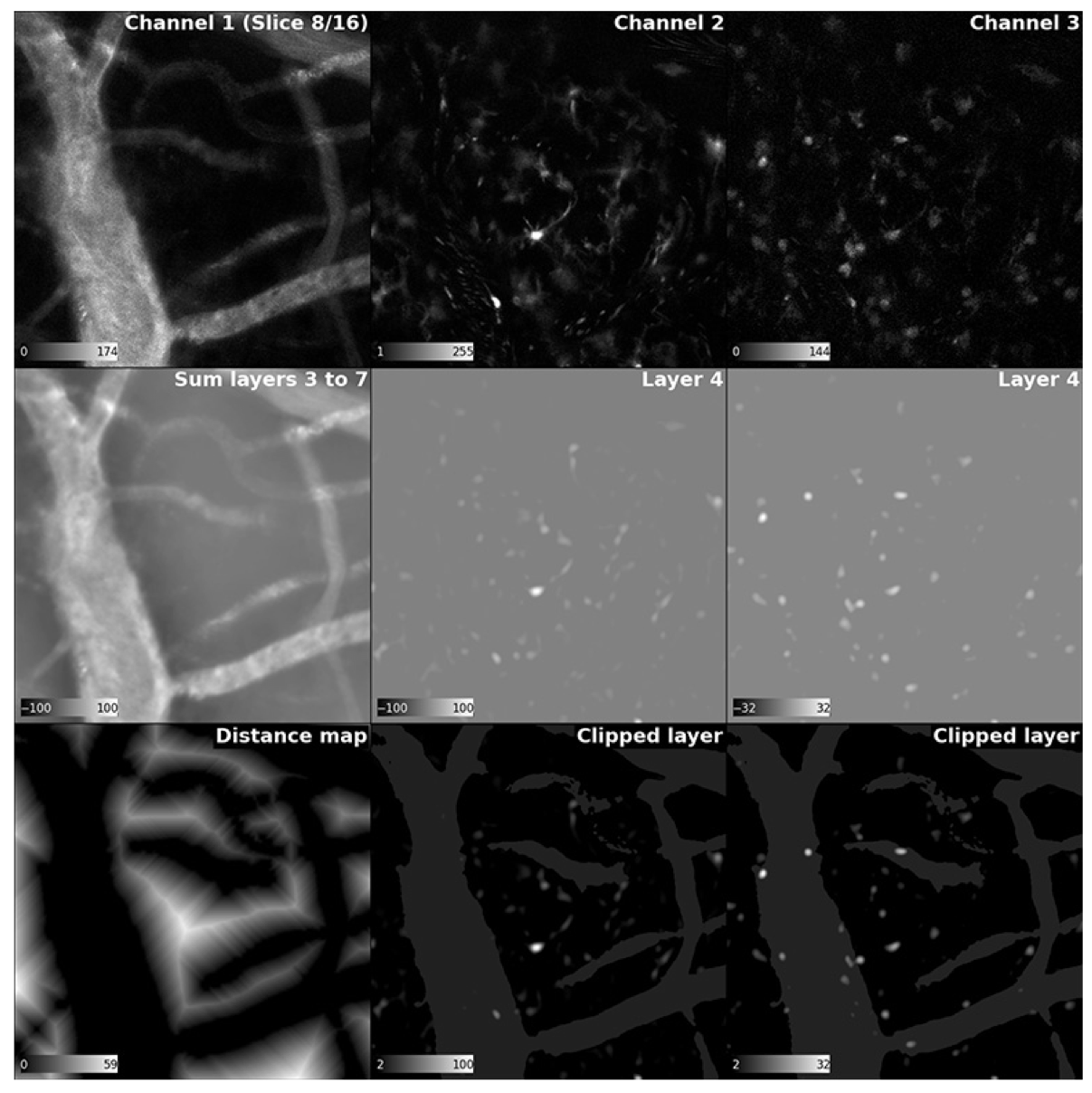

Supplement: S13 Fig — Blood vessels (channel 1) were filtered by summing "A trous" layers 3 to 7 and thresholded to create a binary mask. DsRed T cells (channel 2) and GFP CX3CR1 cells (channel 3) were band-pass filtered using "A trous" layer 4 and pixel values below the set threshold were clipped. Cells within the blood vessel binary mask were ignored for specific tracking of perivascular cells. In the distance map, pixel values are proportional to the distance from the blood vessels and 0 inside the blood vessels. White pixels denote areas furthest from vessels (black pixels). (TIFF) [file ppat.1005210.s013.tiff]
